# A multi-ancestry polygenic risk score improves risk prediction for coronary artery disease

**DOI:** 10.1038/s41591-023-02429-x

**Published:** 2023-07-06

**Authors:** Aniruddh P. Patel, Minxian Wang, Yunfeng Ruan, Satoshi Koyama, Shoa L. Clarke, Xiong Yang, Catherine Tcheandjieu, Saaket Agrawal, Akl C. Fahed, Patrick T. Ellinor, Genes & Health Research Team; the Million Veteran Program, Philip S. Tsao, Yan V. Sun, Kelly Cho, Peter W. F. Wilson, Themistocles L. Assimes, David A. van Heel, Adam S. Butterworth, Krishna G. Aragam, Pradeep Natarajan, Amit V. Khera

**Affiliations:** 1 Division of Cardiology, Department of Medicine, Massachusetts General Hospital, Boston, MA USA; 2 Center for Genomic Medicine, Department of Medicine, Massachusetts General Hospital, Boston, MA USA; 3 Cardiovascular Disease Initiative, Broad Institute of MIT and Harvard, Cambridge, MA USA; 4 Department of Medicine, Harvard Medical School, Boston, MA USA; 5 Cardiovascular Research Center, Massachusetts General Hospital, Boston, MA USA; 6 CAS Key Laboratory of Genome Sciences and Information, Beijing Institute of Genomics, Chinese Academy of Sciences and China National Center for Bioinformation, Beijing, China; 7 Veteran Affairs Boston Healthcare System, Boston, MA USA; 8 Stanford University School of Medicine, Palo Alto, CA USA; 9 Veterans Affairs Palo Alto Healthcare System, Palo Alto, CA USA; 10 Gladstone Institutes, San Francisco, CA USA; 11 Feinberg School of Medicine, Northwestern University, Chicago, IL USA; 12 Veteran Affairs Atlanta Healthcare System, Decatur, GA USA; 13 Blizard Institute, Barts and the London School of Medicine and Dentistry, Queen Mary University of London, London, UK; 14 British Heart Foundation Cardiovascular Epidemiology Unit, Department of Public Health and Primary Care, and Centre of Research Excellence, University of Cambridge, Cambridge, UK; 15 Verve Therapeutics, Boston, MA USA

**Keywords:** Risk factors, Myocardial infarction, Genetics research

## Abstract

Identification of individuals at highest risk of coronary artery disease (CAD)—ideally before onset—remains an important public health need. Prior studies have developed genome-wide polygenic scores to enable risk stratification, reflecting the substantial inherited component to CAD risk. Here we develop a new and significantly improved polygenic score for CAD, termed GPS_Mult_, that incorporates genome-wide association data across five ancestries for CAD (>269,000 cases and >1,178,000 controls) and ten CAD risk factors. GPS_Mult_ strongly associated with prevalent CAD (odds ratio per standard deviation 2.14, 95% confidence interval 2.10–2.19, *P* < 0.001) in UK Biobank participants of European ancestry, identifying 20.0% of the population with 3-fold increased risk and conversely 13.9% with 3-fold decreased risk as compared with those in the middle quintile. GPS_Mult_ was also associated with incident CAD events (hazard ratio per standard deviation 1.73, 95% confidence interval 1.70–1.76, *P* < 0.001), identifying 3% of healthy individuals with risk of future CAD events equivalent to those with existing disease and significantly improving risk discrimination and reclassification. Across multiethnic, external validation datasets inclusive of 33,096, 124,467, 16,433 and 16,874 participants of African, European, Hispanic and South Asian ancestry, respectively, GPS_Mult_ demonstrated increased strength of associations across all ancestries and outperformed all available previously published CAD polygenic scores. These data contribute a new GPS_Mult_ for CAD to the field and provide a generalizable framework for how large-scale integration of genetic association data for CAD and related traits from diverse populations can meaningfully improve polygenic risk prediction.

## Abstract (web summary)

A polygenic risk score for coronary artery disease developed using data from individuals of five different ancestries has increased accuracy across diverse populations.

## Main

Coronary artery disease (CAD) is the leading cause of death worldwide, and identification of at-risk individuals remains a critical public health need^1^. Especially if identified early, at-risk individuals can benefit from more efficiently targeted lifestyle interventions and cholesterol-lowering medications toward lifelong risk mitigation^2^. However, commonly used clinical risk estimators for CAD were optimized for use in middle-aged adult populations in historical cohort studies and consequently underperform in younger populations or individuals of non-European ancestries^3–6^. As CAD is a heritable disease, the increasing amount of widely available genetic data offers additional opportunities to substantially enhance CAD risk prediction early in life, which is likely to prove to be particularly valuable for those in the extremes of the inherited risk distribution^7^.

Polygenic scores integrate data derived from genome-wide association studies (GWASs)—which quantify the relationship between each of many common DNA variants and risk of disease—into a single quantitative and predictive metric of inherited risk. Several studies so far observed substantial gradients in CAD risk, even among participants with similar clinical risk factor profiles, according to a polygenic score^8–11^. Given this potential, polygenic scores are now being deployed clinically across some biobanks and returned through direct-to-consumer testing platforms^12,13^. Although the past decade has seen numerous advances in the predictive capacity of polygenic scores, score performance remains considerably lower than the theoretical maximum, the proportion of trait liability explained by common DNA variants, particularly among individuals of non-European ancestry^14^. Simulation studies suggest that even larger sample sizes of GWASs have the potential to more accurately estimate the effect size associated with each single nucleotide polymorphism to improve scores for CAD^15^. Polygenic scores integrating GWAS data from individuals of diverse ancestries in addition to that of the target population show relative improvement in predictive accuracy compared with methods only utilizing GWAS data from a single ancestry source^16,17^. Furthermore, the principles of genetic correlation suggest benefit in incorporating information from GWAS of related traits to refine polygenic prediction in the trait of interest^18,19^.

Alongside considerable—and warranted—enthusiasm for polygenic scores to enable a new era of preventive clinical medicine is recognition of several key limitations. First, polygenic scores have reduced predictive performance in individuals of non-European ancestry^20^. This largely stems from relative underrepresentation of other ancestries in prior GWAS discovery cohorts. Recent efforts have focused on conducting GWAS in larger and more ancestrally diverse populations and designing methods leveraging ancestry-specific linkage disequilibrium patterns to help improve score performance^16,17,21,22^. Second, although available scores associate strongly with prevalent disease, they perform less well in predicting incident disease, which would offer more clinical utility in enabling targeted interventions^23^. Finally, most risk prediction models so far are based on either genetic or clinical risk factors, but better integration of these modalities and estimation of a clinically actionable risk estimate is needed^24,25^.

In this Article, to address these needs, we used information from ancestrally diverse 269,000 CAD cases, over 1,178,000 controls and data from related traits in over two million individuals along with methods leveraging commonalities in mechanistic pathways to develop a new polygenic risk score for CAD.

## Results

**Fig. 1:**
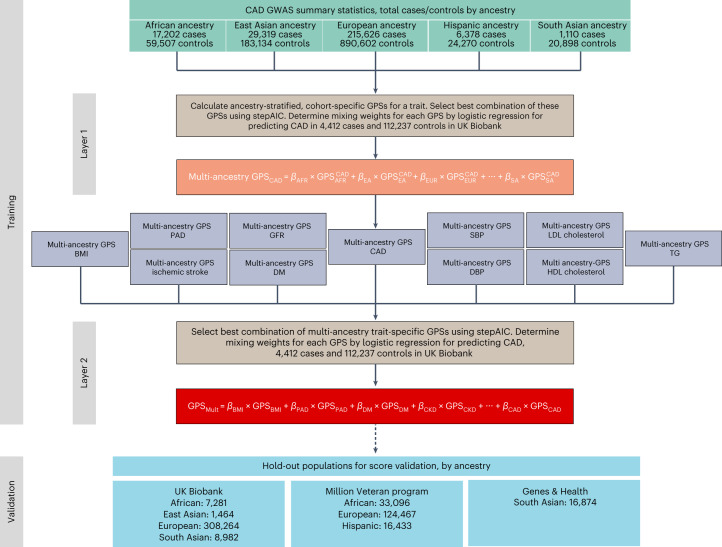
Overview of GPS_Mult_ development. Polygenic scores were constructed using cohort-specific, ancestry-stratified summary statistics for CAD and CAD-related traits, resulting in 51 GPS across all traits and ancestries. For each trait (for example, CAD) the best-performing combination of cohort-specific, ancestry-stratified GPSs was determined using stepAIC, and their optimal mixing weights (*β*) were determined using logistic regression in 116,649 individuals of European ancestry in the UK Biobank training dataset. The selected GPSs were linearly combined using these mixing weights to yield multi-ancestry scores predicting CAD for each trait (layer 1). The best-performing combination of multi-ancestry, trait-specific GPSs was determined using stepAIC, and their optimal mixing weights (*β*) were determined using logistic regression in 116,649 individuals of European ancestry in the UK Biobank training dataset. The selected GPSs were linearly combined using these mixing weights to yield GPS_Mult_ (layer 2). Ancestries: AFR, African; EA, East Asian; EUR, European; HISP, Hispanic; SA, South Asian. Source GWAS traits: CAD^27,33,34,38,56^, body mass index (BMI)^38,57^, ischemic stroke^38,58,59^, diabetes mellitus (DM)^59–61^, peripheral artery disease (PAD)^38,56,62^, glomerular filtration rate (GFR)^38,63^, systolic blood pressure (SBP)^38,64^, diastolic blood pressure (DBP)^38,64^, LDL cholesterol^38,65,66^, HDL cholesterol^38,65,66^, triglycerides (TG)^38,65,66^.

**Fig. 2:**
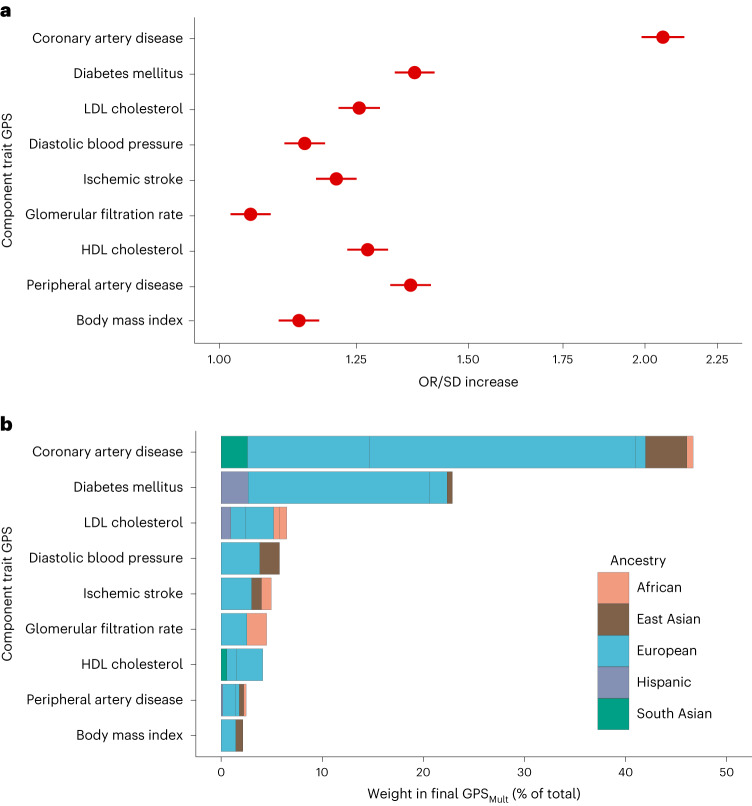
Trait-specific component polygenic score performance and ancestry-specific polygenic score composition of GPS_Mult_. **a**, The OR/SD with 95% CI for prevalent CAD risk of the multi-ancestry, trait-specific layer 1 GPSs was assessed in logistic regression models adjusted for age, sex, genotyping array and the first ten principal components of ancestry in the same training group of *n* = 116,649 independent UK Biobank European ancestry individuals. **b**, The contributing weights of each of the ancestry-stratified, cohort-specific GWAS-based GPS to each of the trait-based layer 1 polygenic scores are proportional to stacked bar size, which are colored according to ancestry of source GWAS, and normalized to 100% to reflect composition in the overall GPS_Mult_. Of 51 ancestry- and trait-specific scores that were included in the GPS training analysis, 32 scores significantly contributed to overall prediction in GPS_Mult_ after optimization of score selection with stepAIC and weighting through logistic regression in the two layers.

Summary statistics from GWAS for CAD, other atherosclerotic diseases (for example, ischemic stroke), and their risk factors (for example, diabetes, blood pressure and lipid concentrations) across over 1.4 million individuals from multi-ancestry cohorts were aggregated to design polygenic risk scores for CAD (Fig. 1 and Supplementary Table 1). These scores were trained within the UK Biobank cohort in 116,649 individuals of European ancestry and then validated in the remaining independent study population of 325,991 individuals (54.3% female, 7281 African, 1,464 East Asian, 308,264 European and 8,982 South Asian ancestry) (Supplementary Table 2)^26^. The participants in the training and validation cohorts are independent from the individuals analyzed in the previously conducted GWAS from which summary statistics were obtained^27^. A total of 51 candidate ancestry- and trait-specific scores were included in the genome-wide polygenic score (GPS) training analysis, with 32 scores carried forward on the basis of a stepwise process to identify those that significantly contributed to overall prediction and included in the weighting of GPS_Mult_ (Fig. 2a,b).

### Association of GPS_Mult_ with prevalent disease in UK Biobank

**Extended Data Fig. 1:**
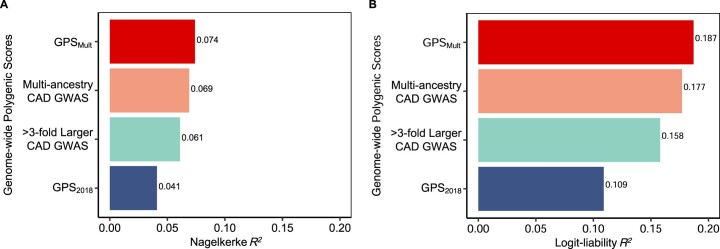
Sequential improvements in *R*^*2*^ with GPS_Mult_ in the UK Biobank Study. The proportion of phenotypic variance explained by the polygenic score predicting coronary artery disease (CAD) was calculated in the UK Biobank European ancestry validation cohort for each GPS score using the A: Nagelkerke’s pseudo-*R*^2^ metric, as the difference of the full model inclusive of the polygenic score plus age, sex, genotyping array, and the first ten principal components of ancestry minus *R*^2^ for the covariates alone; and B: logit-liability *R*^2^ metric. GPS_2018_ denotes previously published polygenic score for CAD^9^. > 3-fold larger CAD GWAS designates metrics for polygenic score generated using summary statistics from the most recent Coronary ARtery DIsease Genome wide Replication and Meta-analysis plus The Coronary Artery Disease Genetics consortium analysis (CARDIOGRAMplusC4D) excluding the UK Biobank. Multi-ancestry CAD GWAS refers to the polygenic score generated by combining ancestry-specific polygenic scores generated using discovery data from Genes & Health, Biobank Japan, Million Veteran Program, FinnGen, and CARDIOGRAMplusC4D (excluding UK Biobank). GPS_Mult_ designates polygenic score for CAD designed with summary statistics from multiple ancestries and multiple CAD-related traits.

**Extended Data Fig. 2:**
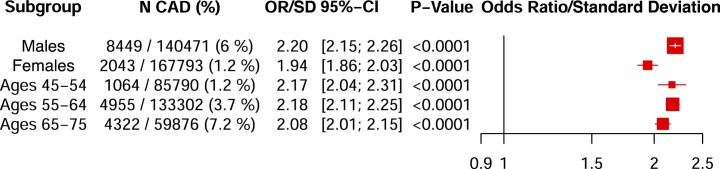
GPS_Mult_ performance by sex and age subgroups. The odds ratio per standard deviation (OR/SD) with 95% confidence intervals for prevalent coronary artery disease (CAD) risk of the GPS_Mult_ was assessed in a logistic regression model adjusted for age, sex, genotyping array, and the first ten principal components of genetic ancestry in the European ancestry validation dataset of the UK biobank (N = 308,264 independent participants) stratified by sex and age subgroups. *P* values are derived from a *t*-test implemented in the GLM function in R and are two-sided.

The resulting score, GPS_Mult_ demonstrated a strong association with prevalent CAD, with significant improvement from previously published scores. Among 308,264 European ancestry individuals in the hold-out validation dataset, GPS_Mult_ was associated with an odds ratio per standard deviation increase (OR/SD) of 2.14 (95% confidence interval (CI) 2.10–2.19) in a model adjusted for age, sex, genotyping array and the first ten principal components of genetic ancestry, with significant improvement from prior published scores from the Polygenic Score Catalog without UK Biobank participants in discovery data, where OR/SD ranged from 1.14 to 1.77 (Supplementary Table 3)^28^. This corresponded to a Nagelkerke *R*^2^ of 0.074 and a logit liability *R*^2^ of 0.187 (Extended Data Fig. 1). After adjusting for measured clinical risk factors including systolic and diastolic blood pressure, low-density lipoprotein (LDL) cholesterol, high-density lipoprotein (HDL) cholesterol, triglycerides, diabetes, body mass index and glomerular filtration rate, this risk estimate was only modestly attenuated to an OR/SD 2.07 (95% CI 2.02–2.13) (Supplementary Table 4). The associations between GPS_Mult_ and CAD were largely consistent across studied subgroups, but some evidence of heterogeneity was found when restricting to male participants (OR/SD 2.20, 95% CI 2.15–2.26, *P* < 0.001) when compared with female participants (OR/SD 1.94, 95% CI 1.86–2.03, *P* < 0.001), with *P*-heterogeneity <0.001 (Extended Data Fig. 2). Additionally, the association between GPS_Mult_ and CAD was stronger in younger individuals aged 45–54 years (OR/SD 2.17, 95% CI 2.04–2.31, *P* < 0.001) and 55–64 years (OR/SD 2.18, 95% CI 2.11–2.25, *P* < 0.001), when compared with older individuals aged 65–75 years (OR/SD 2.08, 95% CI 2.01–2.15, *P* < 0.001), consistent with recent studies (Extended Data Fig. 2)^29,30^.

**Fig. 3:**
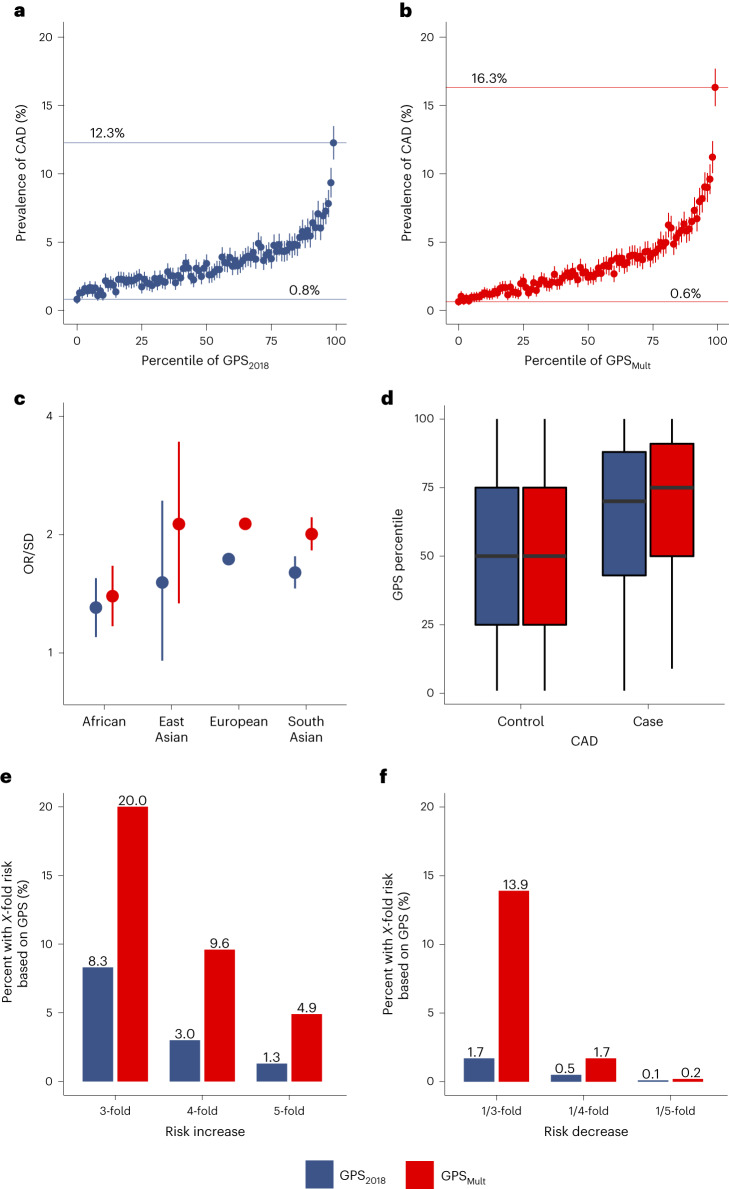
Improvements in polygenic prediction of prevalent CAD prediction. **a**,**b**, The mean prevalence of CAD with 95% CI according to 100 groups of the UK Biobank European ancestry validation dataset consisting of *n* = 308,264 independent participants, binned according to the percentile of the GPS_2018_ (**a**) and GPS_Mult_ (**b**). **c**, The OR/SD with 95% CI for prevalent CAD of GPS_Mult_ was assessed in a logistic regression model adjusted for age, sex and the first ten principal components of ancestry in *n* = 7,281 independent individuals of African ancestry, *n* = 1,464 independent individuals of East Asian ancestry, *n* = 308,264 independent individuals of European ancestry, and *n* = 8,982 independent individuals of South Asian ancestry. **d**, Distributions of GPS_2018_ and GPS_Mult_ percentiles across the UK Biobank European ancestry validation dataset consisting of *n* = 308,264 independent participants. For all box plots: central line of each box, median; top and bottom edges of each box, first and third quartiles; whiskers extend 1.5× the interquartile range beyond box edges. **e**, Proportion of UK Biobank validation population with 3-, 4- and 5-fold increased risk for CAD versus the middle quintile of the population, stratified by GPS. The odds ratio assessed in a logistic regression model adjusted for age, sex, genotyping array and the first ten principal components of ancestry. **f**, Proportion of UK Biobank testing population with 1/3, 1/4, and 1/5 risk for CAD versus the middle quintile of the population, stratified by GPS. Odds ratio assessed in a logistic regression model adjusted for age, sex, genotyping array and the first ten principal components of ancestry.

**Extended Data Fig. 3:**
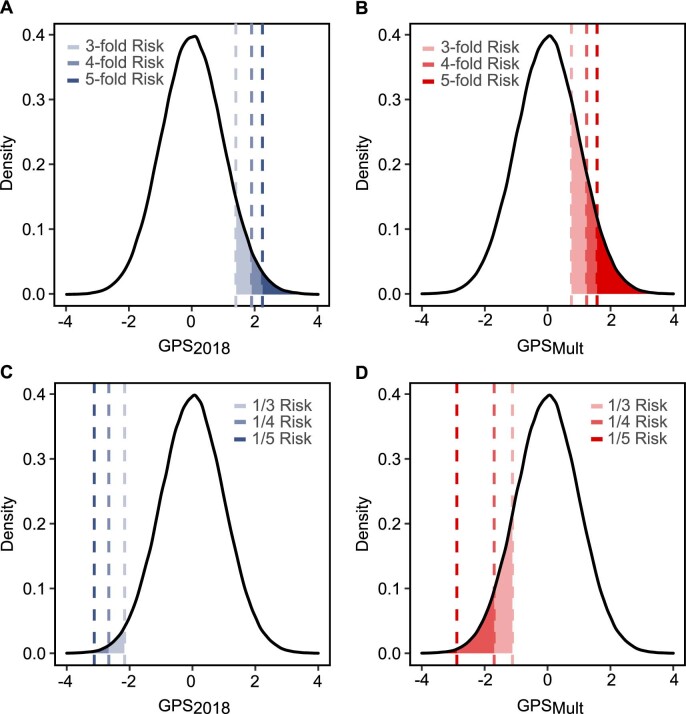
Coronary artery disease risk in the extreme ends of the polygenic score distribution. Proportion of UK Biobank validation population with 3, 4, and 5-fold increased risk for CAD versus the middle quintile of the population identified by GPS_2018_ (A) and GPS_Mult_ (B). The odds ratio assessed in a logistic regression model adjusted for age, sex, genotyping array, and the first ten principal components of ancestry. Proportion of UK Biobank testing population with 1/3, 1/4, and 1/5 risk for CAD versus the middle quintile of the population, population identified by GPS_2018_ (C) and GPS_Mult_ (D). Odds ratio assessed in a logistic regression model adjusted for age, sex, genotyping array, and the first ten principal components of ancestry. GPS: Genome-wide polygenic score; CAD: coronary artery disease.

GPS_Mult_ showed stronger association with CAD risk when compared with the previously published GPS_2018_ (ref. ^9^) in direct comparison using the same group of individuals for validation. Among individuals of European ancestry, individuals in the bottom and top centile of the polygenic score had a 0.8% and 12.3% prevalence of CAD, respectively, with GPS_2018_, compared with 0.6% and 16.3% prevalence of CAD with GPS_Mult_ (Fig. 3 a,b). GPS_Mult_ also outperformed GPS_2018_ in predicting prevalent CAD across ancestry groups in the UK Biobank, with OR/SD of 1.39 (95% CI 1.17–1.67) in African ancestry, 2.14 (95% CI 1.34–3.49) in East Asian ancestry and OR 2.02 (95% CI 1.83–2.23) in South Asian ancestry (Fig. 3c). Among individuals with CAD, the median percentile of GPS_Mult_ is significantly higher than that of the GPS_2018_, 75 (interquartile range 50–91) versus 69 (interquartile range 43–88) (Fig. 3d). Given improved stratification with this newly developed polygenic score, both tails of the score distribution were associated with a greater magnitude of risk when compared with GPS_2018_. With the GPS_2018_, the top 8.3%, 3.0% and 1.3% of the population had 3-fold, 4-fold and 5-fold greater odds for CAD relative to the middle quintile of the population, respectively, whereas with the GPS_Mult_, the top 20.0%, 9.6% and 4.9% of the population had 3-fold, 4-fold and 5-fold greater odds for CAD relative to the middle quintile of the population, respectively (Fig. 3e, Extended Data Fig. 3a,b and Supplementary Table 5). Conversely, with the GPS_2018_, the bottom 1.7%, 0.5% and 0.1% of the population had 1/3, 1/4 and 1/5 the odds for CAD relative to the middle quintile of the population, respectively, whereas with the GPS_Mult_, the bottom 13.9%, 1.7% and 0.2% of the population had 3-fold, 4-fold and 5-fold lower odds of CAD relative to the middle quintile of the population, respectively (Fig. 3f and Extended Data Fig. 3c,d).

### Validation of GPS_Mult_ in external cohorts

**Fig. 4:**
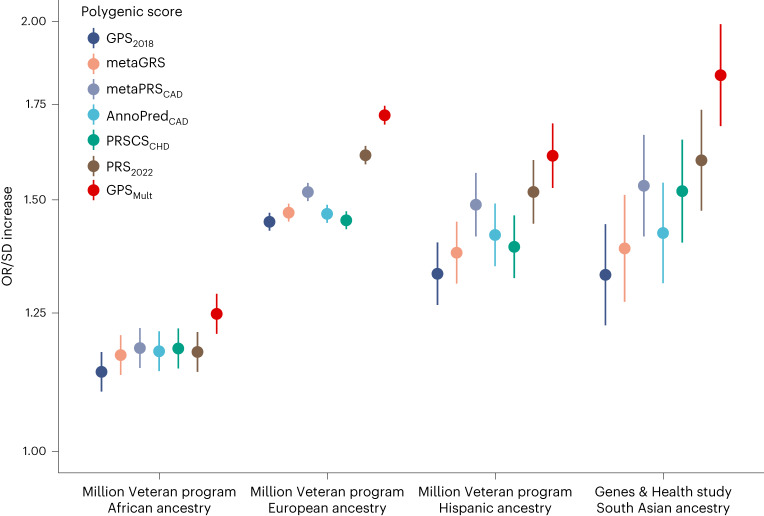
External validation of GPS_Mult_ and benchmarking against published polygenic scores for CAD across multiple ancestries in Million Veteran Program and Genes & Health studies. The OR/SD with 95% CI for prevalent CAD risk was assessed for each polygenic score in a logistic regression model adjusted for age, sex, genotyping array and the first ten principal components of ancestry in the same group of individuals per cohort: *n* = 33,096 independent African ancestry individuals in the Million Veteran Program; *n* = 124,467 independent European ancestry individuals in the Million Veteran Program; *n* = 16,433 independent Hispanic ancestry individuals in the Million Veteran Program; *n* = 16,874 independent South Asian ancestry individuals in the Genes & Health Study, using high-performing published scores from the Polygenic Score Catalog (GPS_2018_ (ref. ^9^), metaGRS^8^, metaPRS_CAD_^67^, AnnoPred_CAD_^68^, PRSCS_CHD_^69^ and PRS_2022_ (ref. ^27^), as well as GPS_Mult_^28^. Results for these and additional CAD polygenic scores published in the Polygenic Score Catalog are available in Supplementary Tables 6 and 7.

GPS_Mult_ was also strongly associated with prevalent CAD in external cohorts, with significant improvement from prior published scores. Twenty-seven polygenic scores for CAD from the Polygenic Score Catalog and GPS_Mult_ were calculated in identical groups of individuals to facilitate direct comparison within individuals of African, European and Hispanic Ancestry in Million Veteran Program^31^ and South Asian ancestry in Genes & Health^32^ (Fig. 4 and Supplementary Tables 6 and 7). For each group, individuals were selected for inclusion that were not included in any of the published GWAS summary statistics^33,34^ used for GPS_Mult_ derivation. Among 33,096 individuals of African ancestry in the Million Veteran Program, GPS_Mult_ was associated with an OR/SD of 1.25 (95% CI 1.21–1.29, *P* < 0.001) for CAD in a model adjusted for age, sex, genotyping array and the first ten principal components of genetic ancestry, corresponding in a 73% (*P* < 0.001) relative improvement in effect size compared with GPS_2018_ and 39% (*P* = 0.008) improvement when compared with the recently published PRS_2022_ (ref. ^27^), respectively. Similarly, among 124,467 individuals of European ancestry in the Million Veteran Program, GPS_Mult_ was associated with an OR/SD of 1.72 (95% CI 1.69–1.75, *P* < 0.001), corresponding in a 46% (*P* < 0.001) and 13.6% (*P* < 0.001) relative improvement in effect size compared with GPS_2018_ and PRS_2022_ (ref. ^27^), respectively. Among 16,433 individuals of Hispanic ancestry in the Million Veteran Program, GPS_Mult_ was associated with an OR/SD of 1.61 (95% CI 1.53–1.70, *P* < 0.001), corresponding in a 66.8% (*P* < 0.001) and 13.9% (*P* = 0.11) relative improvement in effect size compared with GPS_2018_ and PRS_2022_, respectively. Lastly, among 16,874 individuals of South Asian ancestry in Genes & Health, GPS_Mult_ was associated with an OR/SD of 1.83 (95% CI 1.69–1.99, *P* < 0.02), corresponding to a 113% (*P* < 0.001) and 29% (*P* = 0.02) relative improvement in effect size compared with GPS_2018_ and PRS_2022_, respectively (Fig. 4).

### Association of GPS_Mult_ with incident disease in UK Biobank

**Fig. 5:**
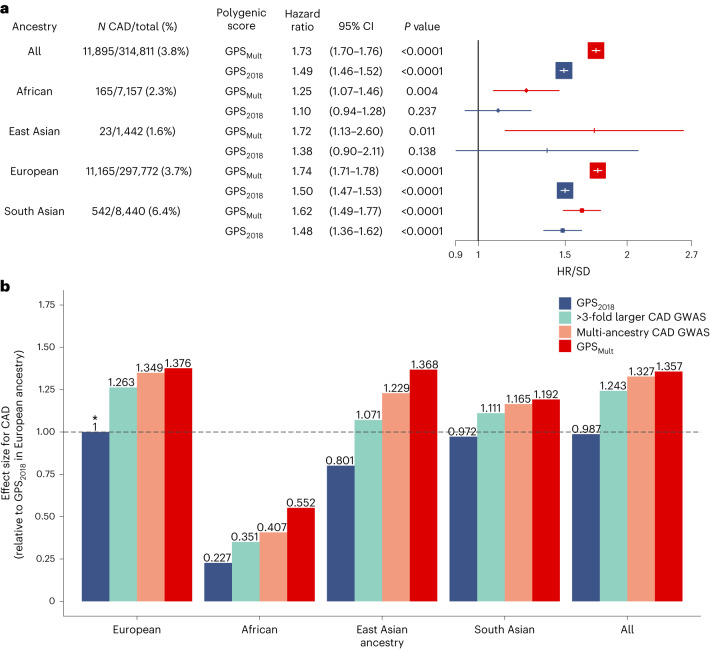
Incident CAD prediction by GPS_Mult_ stratified by ancestry. **a**, Adjusted HR/SD of the polygenic score with corresponding 95% CIs and *P* values for incident CAD by ancestry, stratified by the version of the polygenic score, calculated from Cox proportional-hazards regression models adjusted for age, sex, genotyping array and the first ten principal components of ancestry in the UK Biobank validation dataset, consisting of *n* = 7,157 independent individuals of African ancestry, *n* = 1,442 independent individuals of East Asian ancestry, *n* = 297,772 independent individuals of European ancestry, and *n* = 8,440 independent individuals of South Asian ancestry. GPS_2018_ corresponds to a previously published polygenic score for CAD^9^. *P* values are derived from a Wald test implemented in the coxph function in R and are two-sided. **b**, The score effect sizes relative to the effect size of GPS_2018_ in European ancestry individuals. ‘>3-fold larger CAD GWAS’ designates a polygenic score generated using summary statistics of largely European ancestry from the most recent CARDIOGRAMplusC4D excluding the UK Biobank (GPS_CADEUR_). ‘Multi-ancestry CAD GWAS’ refers to the polygenic score generated by combining ancestry-specific polygenic scores generated using GWAS summary statistics from CARDIOGRAMplusC4D, Genes & Health, Biobank Japan, Million Veteran Program and FinnGEN biobanks in layer 1 (GPS_CADANC_). GPS_Mult_ designates polygenic score for CAD designed with summary statistics from multiple ancestries and multiple CAD-related traits in layer 2. Asterisk designates the reference group for calculating relative gain.

The GPS_Mult_ was predictive of incident CAD events over median (interquartile range) 12.0 (11.2–12.7) years of follow-up across all four ancestral groups in the UK Biobank. Across the entire UK Biobank validation study population without prior CAD, an incident CAD event was observed in 1.1% of those in the lowest percentile of the GPS_Mult_ distribution versus 11.7% of those in the top percentile. Overall, GPS_Mult_ was associated with a hazard ratio per standard deviation (HR/SD) of 1.73 (95% CI 1.70–1.76, *P* < 0.001), compared with hazard ratio (HR) 1.49 (95% CI 1.46–1.52, *P* < 0.001) found with GPS_2018_. When stratified by ancestry, risk estimates were comparable across individuals of East Asian (HR/SD 1.72, 95% CI 1.13–2.60, *P* = 0.011), European (HR/SD 1.74, 95% CI 1.71–1.78, *P* < 0.001), and South Asian (HR/SD 1.62, 95% CI 1.49–1.77, *P* < 0.001) ancestry, but effect size was reduced among individuals of African ancestry (HR/SD 1.25, 95% CI 1.07–1.46, *P* = 0.004) (Fig. 5a). Across all individuals in the UK Biobank validation dataset, GPS_Mult_ demonstrated 38% relative improvement in effect size compared with GPS_2018_. Of this, 26% improvement resulted from larger sample size of the primary Coronary ARtery DIsease Genome wide Replication and Meta-analysis plus The Coronary Artery Disease Genetics consortium (CARDIOGRAMplusC4D) GWAS (excluding UK Biobank participants), 9% improvement from incorporation of multi-ancestry CAD summary statistics, and 3% improvement from leveraging genetic commonalities with CAD risk factors to refine score weighting (Fig. 5b). Incorporation of multi-ancestry and multi-trait genetic data resulted in greater relative gains in incident disease prediction for individuals in each ancestry, with improved relative effect sizes of 143%, 71%, 38% and 23% for individuals of African, East Asian, European and South Asian ancestry, respectively, compared with GPS_2018_ performance in those groups. Enhanced performance, indexed to the effect size observed in European ancestry with the GPS_2018_, was also observed across ancestries, with improved prediction in African ancestry (relative effect size 0.55, increased from 0.23) (Fig. 5b) and performance surpassing the reference score in East Asian ancestry (relative effect size 1.37, increased from 0.80) and South Asian ancestry (relative effect size 1.19, increased from 0.97).

### Disease risk in the extremes of the GPS_Mult_ distribution

**Extended Data Fig. 4:**
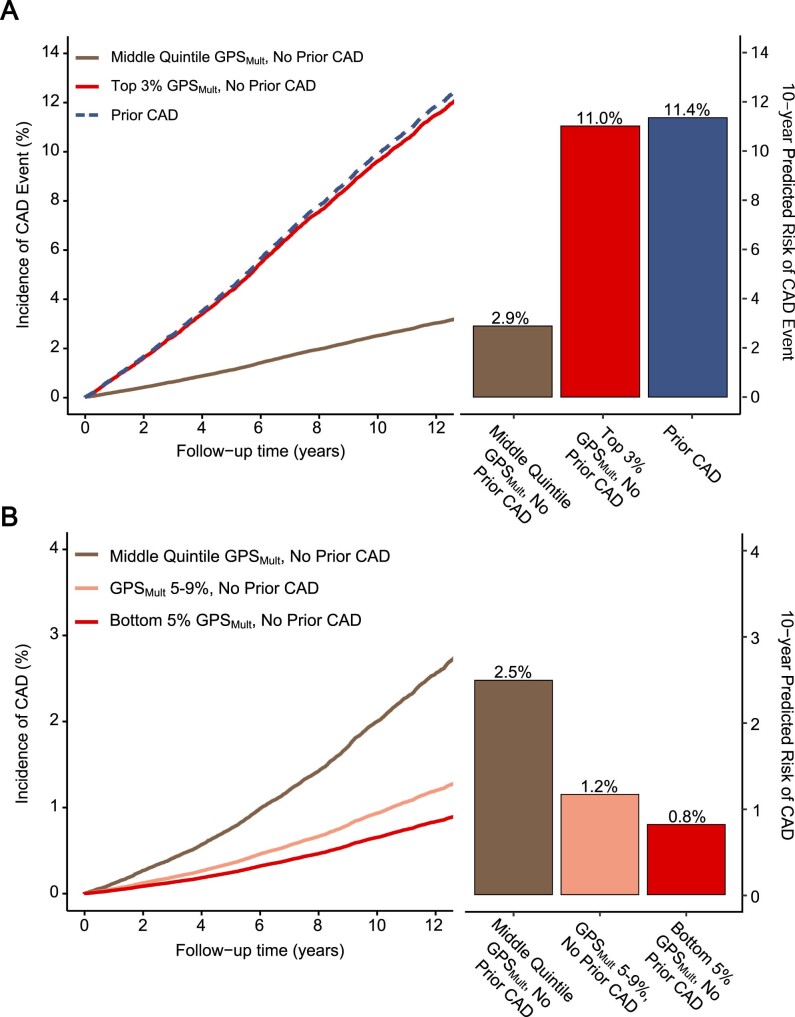
Extremes of risk for incident coronary artery disease identified by tail distributions of GPS_Mult._ A: Cumulative incidence of coronary artery disease (CAD) events (%) over length of the follow-up period stratified by presence of prior CAD or with no prior CAD and for the middle quintile or top 3% of the population for GPS_Mult_ risk, estimated using Cox proportional-hazards regression model adjusted for age, sex, genotyping array, and the first ten principal components of ancestry in the European ancestry UK Biobank validation dataset. The estimated 10-year CAD event risk was predicted using same model standardized to the mean of each of the covariates. B: Cumulative CAD risk (%) stratified by the bottom 5%, the 5–9% segment, and the 40–59% segment of the population for GPS_Mult_ risk, estimated using Cox proportional-hazards regression model adjusted for age, sex, genotyping array, and the first ten principal components of ancestry in the European ancestry validation dataset of the UK Biobank Study. The estimated 10-year CAD risk was predicted using same model standardized to the mean of each of the covariates. GPS: Genome-wide polygenic score.

**Extended Data Fig. 5:**
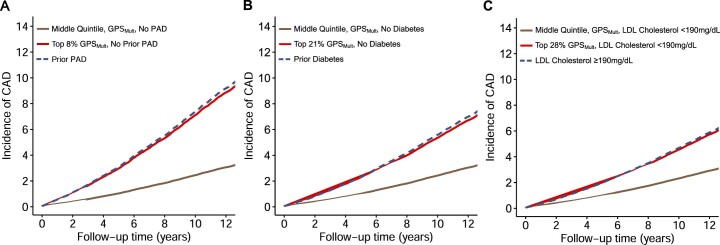
Equivalents of increased risk for incident coronary artery disease event identified by high GPS_Mult_ in the UK Biobank Study. A: Cumulative incidence of coronary artery disease (CAD) events (%) over length of follow-up stratified by presence of prior peripheral artery disease (PAD) or no prior PAD with GPS_Mult_ in the middle quintile or top 8% of the population, estimated using Cox proportional-hazards regression model adjusted for age, sex, genotyping array, and the first ten principal components of ancestry in the European ancestry UK Biobank validation dataset. B: Cumulative incidence of CAD events (%) over length of follow-up stratified by presence of prior diabetes mellitus (DM) or no prior DM with GPS_Mult_ in the middle quintile or top 21% of the population, estimated using Cox proportional-hazards regression model adjusted for age, sex, genotyping array, and the first ten principal components of ancestry in the European ancestry UK Biobank validation dataset. C: Cumulative incidence of CAD events (%) over length of follow-up stratified by presence of prior severe hypercholesterolemia (estimated untreated low-density lipoprotein cholesterol, LDL-C 190 mg/dL or higher), or no prior hypercholesterolemia with GPS_Mult_ in the middle quintile or top 28% of the population, estimated using Cox proportional-hazards regression model adjusted for age, sex, genotyping array, and the first ten principal components of ancestry in the European ancestry UK Biobank validation dataset.

We additionally hypothesized that the GPS_Mult_ could identify individuals in the extreme tails of its distribution with clinically important increase, or decrease, in risk. Current cardiovascular disease prevention guidelines recommend statin therapy for individuals with prior CAD, peripheral artery disease (PAD), ischemic stroke, diabetes mellitus or severe hypercholesterolemia (LDL ≥190 mg/dL to help mitigate their high risk of cardiovascular disease and mortality^2^. In the high end of GPS_Mult_, we sought to identify individuals with genetic risk of equivalent magnitude to that of individuals with these guideline-endorsed indications for statin therapy. In prospective analyses of individuals without prior CAD, when compared with individuals in the middle quintile, those within the top 3 percentiles of GPS_Mult_ had equivalent disease risk of incident CAD as the recurrent event risk for an individual who had a CAD event before enrollment (Extended Data Fig. 4a). Furthermore, individuals without PAD in the top 8% of polygenic score distribution had incident CAD risk equivalent to individuals with prior PAD; individuals without diabetes in the top 21% of polygenic score distribution had incident CAD risk equivalent to individuals with prior diabetes; and individuals without severe hypercholesterolemia (estimated untreated LDL cholesterol ≥190 mg/dL) in the top 28% of polygenic score distribution had incident CAD risk equivalent to individuals with prior hypercholesterolemia (Extended Data Fig. 5a–c). Conversely, in the low end of the GPS_Mult_ distribution, individuals in the bottom 5 percentiles were associated with a significant reduction in incident CAD risk (HR 0.27, 95% CI 0.21–0.35, *P* < 0.001) when compared with the middle quintile (40–59%). When comparing individuals who smoke and are in the bottom 5 percentiles of GPS_Mult_ with nonsmokers in the middle quintile, the reduction in the absolute incidence of CAD associated with low GPS_Mult_ offsets approximately 60 pack-years of smoking. Furthermore, individuals in the 5th to 9th percentiles of GPS_Mult_ also had a significant reduction in CAD risk (HR 0.55, 95% CI 0.49–0.62, *P* < 0.001) when compared with the middle quintile. These individuals experienced comparable risk reduction as those individuals carrying variants in *PCSK9*-associated lifelong low levels of LDL cholesterol (Extended Data Fig. 4b)^35,36^.

### Modeling of GPS_Mult_ with clinical risk predictors

**Fig. 6:**
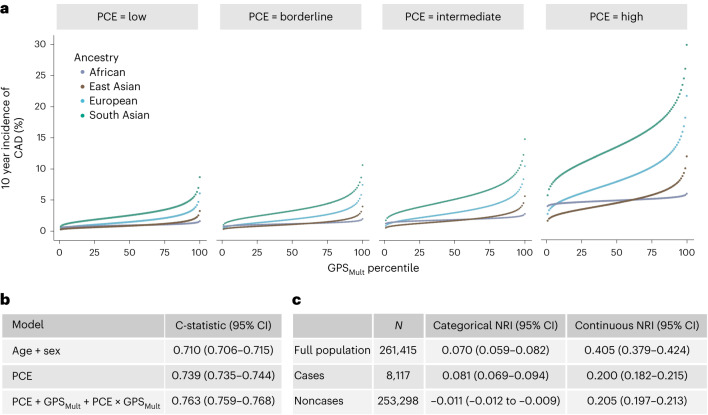
Discrimination and reclassification by a model integrating polygenic and clinical risk for incident CAD. **a**, The cumulative incidence of CAD over 10 years predicted by modeling GPS_Mult_, AHA/ACC PCE 10-year risk estimate, and their interaction in the UK Biobank validation dataset binned according to the percentile of the GPS_Mult_. Individuals were grouped by risk categories of the PCE (predicted 10-year risk of atherosclerotic cardiovascular disease as ‘low’ (<5%), ‘borderline’ (5% to <7.5%), ‘intermediate’ (≥7.5% to <20%) and ‘high’ (≥20%)), and stratified by ancestry. **b**, C-statistics are based on 10-year follow-up events from Cox regression models of listed variables. PCE includes age and sex variables in its risk estimation. **c**, The improvement in the predictive performance of the addition of the GPS_Mult_ to the PCE was evaluated using continuous and categorized NRI, with a risk probability threshold of 7.5% and CIs (95%) obtained from 100-fold bootstrapping.

A risk prediction approach integrating clinical and genetic risk using the American College of Cardiology/American Heart Association Pooled Cohort Equations (PCE)^5^, GPS_Mult_ and their interaction in a single model was used to predict 10-year risk of CAD in the UK Biobank validation population. Accounting for the interaction between the polygenic score and clinical risk estimate improves performance beyond the simple addition of the two, with lower GPS_Mult_ weighting with higher PCE estimates (interaction effect size −0.60, *P*_interaction_ < 0.001). This combined model effectively improved risk prediction when compared with PCE alone. When binned into strata corresponding to clinical guideline recommendations^5^, this model suggested striking gradients in predicted CAD incidence across the GPS_Mult_ distribution, with significant differences observed in ancestry-based subgroups (Fig. 6a). The absolute gradient in risk predicted by this model from bottom to top centile was largest in South Asian ancestry individuals with high PCE risk (5.1% to 29.1%), compared with European ancestry individuals (2.6% to 20.6%).

**Extended Data Fig. 6:**
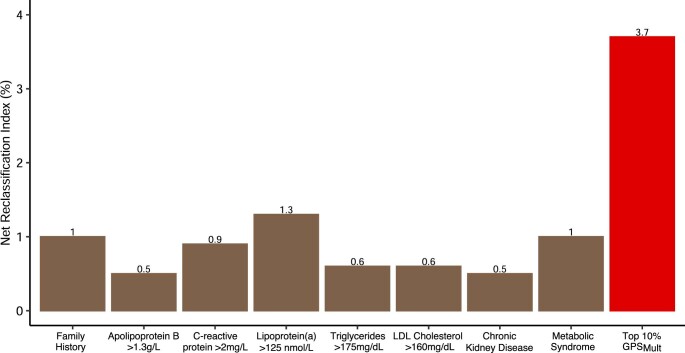
Net reclassification with presence of high GPS_Mult_ or CAD risk enhancing factors over PCE 10-year risk estimates. Net reclassification of coronary artery disease (CAD) cases and non-cases at the 7.5% threshold achieved by presence of established CAD risk enhancing factors or high GPS_Mult_ when added to a baseline model of just the American Heart Association/American College of Cardiology Pooled Cohort Equations^5^ in the European ancestry validation dataset of the UK Biobank.

When compared with the PCE risk estimate incorporating clinical risk factors alone, integration of the PCE with GPS_Mult_ contributed to significantly higher discrimination and predictive performance across the entire tested population. First, discrimination was assessed in Cox regression models including various covariables using Harrell’s C-statistic. A gradient in improvement was seen using baseline models with age and sex alone (C-statistic 0.710, 95% CI 0.706–0.715), PCE, which is inclusive of age and sex (C-statistic 0.739, 95% CI 0.735–0.744), and the model integrating PCE, GPS_Mult_ and their interaction term (C-statistic 0.763, 95% CI 0.759–0.768) (Fig. 6b). Similar improvements in C-statistic were observed for models tested in subgroups stratified by ancestry (Supplementary Table 8). Second, categorized net reclassification improvement (NRI) was calculated across the entire study population using a threshold of 7.5% (NRI 0.075) of the predicted 10-year risk of CAD, which is the clinically accepted estimated risk threshold for recommending initiation of statin therapy for prevention of CAD. The risk model combining PCE and GPS_Mult_ resulted in significant improvements in the categorical net reclassification index (NRI 7.0%, +8.1% for incident cases and −1.1% for noncases), with GPS_Mult_ resulting in greater up classification of risk largely in individuals who go on to develop disease (Fig. 6c). Third, when compared with established risk-enhancing factors for CAD, categorization within the top 10 percentiles of the GPS_Mult_ distribution corresponded to a significantly higher net reclassification over the use of PCE estimate alone (3.7%) as compared with other risk enhancers such as elevated lipoprotein (a) (with NRI 1.3%) (Extended Data Fig. 6). Similar results in NRI were observed across other ancestries (Supplementary Table 9). Additionally, similar trends in predictive performance, discrimination and reclassification were observed in a model that included integration of the QRISK clinical risk estimator, instead of the PCE, with GPS_Mult_ (Supplementary Tables 8 and 9).

### Association of GPS_Mult_ with recurrent disease in UK Biobank

**Extended Data Fig. 7:**
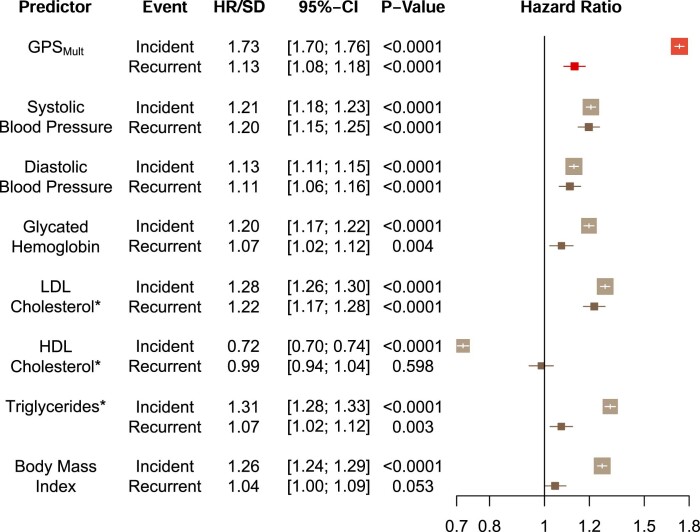
Association of GPS_Mult_ and risk factors with incident and recurrent coronary artery disease events. Hazards ratio per standard deviation (HR/SD) with 95% confidence intervals of variable of interest for incident disease assessed in individuals without prior coronary artery disease (CAD) followed for development of first CAD event with Cox proportional hazards regression model adjusted for age, sex, genotyping array, and the first ten principal components of ancestry in the European ancestry validation dataset of the UK Biobank Study (N = 308,264 independent participants). HR/SD of variable of interest for recurrent disease assessed in individuals with prior CAD followed for development of recurrent CAD event with Cox proportional hazards regression model adjusted for age, sex, genotyping array, and the first ten principal components of ancestry. *P* values are derived from a Wald test implemented in the coxph function in R and are two-sided. *LDL-C, HDL-C, and triglyceride values were adjusted for cholesterol-lowering medication status, as previously described^57^. BP: Blood pressure. BMI: Body-mass index. HgbA1c: Glycated hemoglobin. LDL-C: Low-density lipoprotein cholesterol. HDL-C: High-density lipoprotein cholesterol.

In addition to first events, the GPS_Mult_ predicted recurrent CAD events in individuals with prior CAD. GPS_Mult_ was associated with an HR/SD of 1.13 (95% CI 1.08–1.18, *P* < 0.001), comparable to prior studies^37^. Although a significantly less pronounced effect estimate as compared with the prediction of a first CAD event, the predictive performance of GPS_Mult_ in this context was comparable to that of diastolic blood pressure (HR 1.11, 95% CI 1.06–1.16, *P* < 0.001) and glycated hemoglobin (HR 1.07, 95% CI 1.02–1.12, *P* < 0.001) (Extended Data Fig. 7).

## Discussion

A new polygenic score for CAD incorporating multi-ancestry summary statistics from GWAS for CAD and related risk factor traits on a large scale demonstrated significantly improved performance when compared to prior published scores. External validation in fully independent datasets derived from the Million Veteran Program and the Genes & Health studies confirmed enhanced prediction compared withpreviously published and available polygenic scores across multiple ancestries. The enhanced predictive capacity of this score was particularly pronounced in the extremes of the score distribution, enabling—in some cases—identification of healthy individuals with risk of CAD equivalent to those with pre-existing disease. When added to risk scores used in current clinical practice, GPS_Mult_ significantly improved discrimination and reclassification relevant to clinically important decision thresholds, such as the decision to initiate statin therapy.

This work builds on prior studies in providing a framework for optimizing a polygenic score for any trait, within the limitations of available GWAS with finite sample sizes and underrepresentation of diverse populations. The GPS_Mult_ incorporates CAD summary statistics from large non-European ancestry biobanks encompassing over 269,000 cases and over 1,178,000 controls, including many-fold larger representation of individuals of non-European ancestries than previously published efforts^32,33,38,39^. This results in substantial improvements in prediction for individuals of East and South Asian ancestry, reflecting greater representation of summary statistics from Biobank Japan and Genes & Health. However, the majority of improvement in effect size is attributable to use of summary statistics from the largest CAD GWAS so far (CARDIOGRAMplusC4D consortium, excluding UK Biobank participants), particularly in European ancestry individuals^27^. The additional incorporation of genetic associations with CAD-related risk factors across ancestries into calculating GPS_Mult_ significantly improves prediction beyond using summary statistics from CAD GWAS alone, with impact most notable in individuals of non-European ancestry. This may potentially be due to greater representation of these ancestries in the discovery GWAS for CAD risk factor traits. With these additions, the phenotypic variance explained by GPS_Mult_ for CAD calculated as *R*^2^ on the logit-liability scale was 0.187. Although this estimate remains below the estimated single nucleotide polymorphism heritability for CAD of 0.4–0.6, it surpasses the phenotypic variance explained of 0.155 by the largest component GWAS from the CARDIOGRAMplusC4D consortium^27,40^.

Overall, modest improvements in prediction were observed among individuals of African ancestry, in part due to underrepresentation of this group in GWASs so far, and these discrepancies warrant careful consideration as polygenic scores start to enter into clinical practice^20^. Due to smaller haplotype blocks observed in individuals of African ancestry, a 4- to 7-fold larger GWAS is needed to yield comparable prediction gains^41^. In the near term, the decreased effect size observed in individuals of African ancestry is likely to persist, and this has also been observed for other biomarkers and predictors in clinical practice^42^. Nevertheless, genetic ancestry has a considerable impact on certain aspects of polygenic risk prediction, such as the allele frequency of a given variant. In order to best to move polygenic scores into widespread practice, research efforts would benefit from transparent and systematic reporting of score performance across ancestries^43^, recruitment of more diverse study participants in cohorts such as the US AllofUs Research Program^21^, new statistical methods to enhance cross-ancestry portability^16,17^, more sophisticated quantitative metrics and ongoing dialog with a range of stakeholders, including patients^44^. Furthermore, as the population of admixed individuals that do not discretely mapping onto a single continental ancestry continues to increase, recently developed methods that account for more continuous representations of ancestry in polygenic scores may prove useful^45^.

Polygenic scores have the potential to enhance clinical decision making, although this warrants confirmation in prospective studies. Some such studies are already underway returning polygenic risk information to patients^12,46^, and medical societies have begun to provide provisional guidance on their use^47^. Furthering these goals, GPS_Mult_ is able to better identify individuals at the highest risk for developing incident CAD to potentially guide early preventive interventions^48,49^. Building on prior work advocating for use of polygenic scores as a risk-enhancing factor to guide decision making regarding statin therapy in individuals at borderline or intermediate CAD risk, the current work more strongly supports use in primary screening across the population to target interventions^50^. Current cardiovascular prevention guidelines recommend statin initiation for individuals solely on the basis of having any of the following conditions as they portend high risk of a new atherosclerotic cardiovascular disease event: prior CAD, ischemic stroke, PAD, diabetes or severe hypercholesterolemia^2^. Here we demonstrate that GPS_Mult_ identified 3% of the population with equivalent risk for a future CAD event as that in individuals who have had prior disease. Similarly, the top 8%, 21% and 28% of the GPS_Mult_ distribution—despite having no known CAD—had equivalent risk of incident CAD as individuals with prior PAD, diabetes mellitus and severe hypercholesterolemia, respectively. Because all three of these designations are currently clinical indications for statin therapy, a high GPS_Mult_ could be employed to identify additional individuals for cholesterol-lowering therapies as an adjunct to current guidelines. Furthermore, given the GPS_Mult_’s ability to identify these individuals with the highest propensity for developing CAD, these scores could be employed to enrich for high-genetic-risk individuals in CAD prevention trials to maximize event rates and minimize drug trial costs^51^. The GPS_Mult_ could also be employed to identify the individuals with the highest risk of recurrent events for targeted, otherwise costly therapies that have been shown to be beneficial in this population^52,53^. Additionally, GPS_Mult_ also identifies individuals in the lower end of genetic risk who are seemingly protected from CAD with similar risk reduction as that of carriers of variants in the *PCSK9* gene leading to lifelong reductions on LDL cholesterol^35,36^.

Furthermore, a risk model incorporating polygenic risk with the PCE estimated risk is applied to individuals across different ancestries to demonstrate improved predictive performance. This improved performance illustrates the potential for an integrated absolute risk prediction model^24,25^. For example, this model is particularly useful in differentiating risk in the high-risk South Asian ancestry population, where traditional clinical risk estimators often fail to capture the increased risk associated with this ancestry^4^. The integration of the GPS_Mult_ with PCE builds on prior efforts that demonstrated improvement in model discrimination by now showing nearly identical improvement in C-statistic (0.03) in between models incorporating (1) age and sex, (2) PCE alone and (3) combined genetic and clinical risk across the population^23^. However measures of C-statistic alone are not optimal or fully comprehensive in evaluating models that predict future risk^54^. GPS_Mult_ demonstrates nearly three-fold greater net reclassification of CAD cases/noncases when added to the PCE 10-year risk assessment to guide statin initiation as compared with established ‘risk-enhancing factors’. Further work is needed to incorporate additional risk factors. To aid in future model calibration efforts, there is a need for population-level disease incidence and mortality data disaggregated by ancestral subgroups^12^.

These results should be interpreted within the context of limitations. Polygenic scores were developed and validated in individuals of European ancestry and then externally validated in non-European ancestry populations, and this may have contributed to decreased predictive performance in these groups. These results underscore the need for larger and more representative GWAS studies. UK Biobank participants were recruited at age 40–69 years, raising the possibility of survivorship or selection bias that limits generalizability to younger patients; however, recent studies have demonstrated reliable performance of GPS in younger age groups^7^. All UK Biobank disease endpoints were similarly ascertained through participant self-report, diagnosis codes from inpatient admissions, national procedure, and death registries. Relatively few incident events were observed in individuals of non-European ancestry in the UK Biobank, and additional work is needed to evaluate this in larger populations and further validate optimal approaches to integrate GPS with clinical risk scores. Participants in research studies tend to be healthier than the general population—recalibration of disease risk models for a given target population may be needed before clinical deployment^55^.

In conclusion, incorporating GWAS data for CAD and related traits from multiple ancestries on a large scale leads to significantly improved performance of GPS_Mult_ in external validation among diverse ancestry populations when compared with previously published scores. This approach is readily generalizable to common complex diseases and traits, results in a polygenic score that is able to better identify individuals at the highest and lowest ends of risk, significantly reclassifies risk beyond clinical risk estimators, and has the potential to advance clinical decision making.

## Methods

### Study populations

The UK Biobank is a prospective cohort study that enrolled over 500,000 individuals between the ages of 40 and 69 years between 2006 and 2010 (refs. ^26,70^). A detailed questionnaire completed by UK Biobank participants at enrollment assessed self-report of sex, ancestry and lifestyle factors, including smoking. Anthropometric measurements including body mass index were measured at the initial enrollment visit. Biomarkers including serum lipid concentrations and renal function markers were assessed at time of enrollment as part of the study protocol. Diagnoses of PAD, diabetes and hypertension were determined on the basis of self-report, hospitalization records, procedure codes and death registry codes confirming a clinical diagnosis^4,71^.

Participants within the Million Veteran Program were recruited from more than 75 Veteran Affairs Medical Centers nationwide since 2011, with >885,000 individuals currently enrolled^31^. Each participant has consented to linkage to their electronic medical record, wherein self-reports of ancestry and sex, ICD9/10 diagnosis codes, Current Procedural Terminology codes, clinical laboratory measurements and reports of diagnostic imaging modalities are available. Participants were also asked to complete baseline and lifestyle questionnaires to further augment data contained in the electronic health record.

Genes & Health is a UK-based cohort of over 48,000 British Pakistani and Bangladeshi individuals recruited and consented for lifelong electronic health record access and genetic analysis^32^. Medical records are linked to ICD10, OPCS and SNOMED diagnosis and procedural codes across inpatient and hospital settings as well as clinical laboratory measurements, and a baseline questionnaire containing demographic information including self-report of sex and ancestry.

### Clinical endpoints

Ascertainment of CAD at enrollment in the UK Biobank was based on self-report, hospitalization records, procedural codes or death registry confirming diagnosis of myocardial infarction or its acute complications, or a coronary revascularization procedure (coronary artery bypass graft surgery or percutaneous angioplasty/stent placement)^71,72^. The earliest date at which the diagnosis was ascertained was considered as the diagnosis date. For individuals with CAD before enrollment, recurrence of CAD was determined on the basis of diagnosis of a myocardial infarction or revascularization in the follow-up period after study enrollment^73^.

Within the Million Veteran Program, ICD9, ICD10 and Current Procedural Terminology codes from both inpatient and outpatient encounters were used to curate and classify CAD cases based on having a myocardial infarction or undergoing revascularization, identified as subjects with at least two codes (of any category) that occurred on distinct dates within a 12 month window^33^. Incident cases were identified as those with the first of the two qualifying codes occurring after enrollment. The remaining CAD cases, including through self-report, were considered prevalent.

In the Genes & Health study, ICD10 and SNOMED codes from the linked electronic health record were used to classify CAD cases defined as myocardial infarction or revascularization on the basis of first diagnosis date^34^. Prevalent cases were defined as events before enrollment while events occurring after enrollment were designated as incident disease.

### GPS construction

Summary statistics from recent CAD GWAS studies (Genes & Health, FinnGen, Million Veteran Program, Biobank Japan and CARDIOGRAMplusC4D excluding UK Biobank samples) conducted in individuals of diverse ancestries were used to determine primary CAD score weights (Supplementary Table 1)^27,32,33,38,39^. UK Biobank participants were not included among these discovery cohorts to preserve them as an independent hold-out dataset for training and validation of the GPS_Mult_ (Supplementary Table 2). Ancestry-specific linkage disequilibrium reference panels were extracted from the 1000 Genomes Project phase 3 data to match with the ancestry for the discovery GWAS, and only unrelated samples were used^74^. GPS_Mult_ construction comprised a two-layer process, with layer 1 consisting of combining multiple polygenic scores derived from different ancestry-specific GWAS data for each trait, and layer 2 consisting of combining this multi-ancestry CAD polygenic score with similarly constructed multi-ancestry CAD-related trait scores predicting CAD (Fig. 1) to generate GPS_Mult_.

Separate GPS were constructed for each ancestry-stratified CAD GWAS using the LDpred2 method, which is a Bayesian approach to calculate a posterior mean effect for all variants based on an effect size in the prior GWAS and subsequent shrinkage based on linkage disequilibrium^75^. Only HapMap3 variants—a set of 1,296,172 variants compiled by the International HapMap Project which capture common patterns of variation in a variety of human populations—were included for score calculation^76^. The default parameters used in the LDpred2 method included the proportion of variants assumed to be causal (cut-offs of *P* = 1.0 × 10^−4^, 1.8 × 10^−4^, 3.2 × 10^−4^, 5.6 × 10^−4^, 1.0 × 10^−3^, 1.8 × 10^−3^, 3.2 × 10^−3^, 5.6 × 10^−3^, 1.0 × 10^−2^, 1.8 × 10^−2^, 3.2 × 10^−2^, 5.6 × 10^−2^, 1.0 × 10^−1^, 1.8 × 10^−1^, 3.2 × 10^−1^, 5.6 × 10^−1^ and 1), the scale of heritability (*s* = 0.7, 1 and 1.4) and whether or not a sparse LD matrix was applied^9,75,77^. Combinations of these parameters resulted in 102 candidate GPSs for each set of ancestry-stratified GWAS summary statistics. We extracted the genotypes from centrally imputed data repository, manipulated and transformed the data by bgenix and BCFtools^78,79^, computed the polygenic scores by the Plink software parallelly for each chromosome, and combined the chromosome scores for each individual by the Datamash software^80,81^. The best GPS was selected among these candidates by assessing their performance in predicting prevalent CAD in an independent 116,649 individuals of White British ancestry from UK Biobank (this dataset was used in all the score selection procedures thereafter, and same group of individuals used to train previously published score GPS_2018_ who had not withdrawn consent in the interim)^9^. For example, using the GWAS data from CARDIOGRAMplusC4D excluding UK Biobank samples, the best-performing score predicting CAD (GPS_CADEUR_) was generated using LDpred2 parameters of *P* = 0.018, *h*^2^ scale = 1, and without sparse LD. For selecting the best combination of CAD GPS scores from each ancestry-specific CAD GWAS for mixing, the discriminative capacities (Akaike information criterion, AIC) of these GPS combinations for predicting CAD were assessed using the stepAIC function from R MASS package^82^. A logistic regression model was used to estimate the mixing weights for each individual ancestry-specific GPS. These GPSs were then linearly combined together into a single GPS_CADANC_ score (layer 1, Fig. 1). Similar procedures were followed for other atherosclerotic diseases (ischemic stroke and PAD)^58,62^ and risk factor traits—LDL cholesterol, HDL cholesterol, triglycerides^65,66^, diabetes^60^, systolic blood pressure^83^, diastolic blood pressure, glomerular filtration rate^63^ and body mass index (Supplementary Table 1 and Fig. 1).

These multi-ancestry trait-specific GPSs were then linearly combined with the multi-ancestry GPS_CADANC_ (from layer 1) to generate the final GPS_Mult_ (layer 2). Just as for layer 1, the discriminative capacities (AIC) of these GPS combinations for predicting CAD were assessed to identify the best combination of trait-level scores for mixing^82^. A logistic regression model was used to estimate the mixing weights for each individual trait-specific GPS as described above. These GPSs were then linearly combined together into a single GPS_Mult_ score (layer 2, Fig. 1). Of 51 GWAS- and ancestry-specific GPS that went through layers 1 and 2 of selection and mixing, 32 contributed to the final GPS_Mult_, incorporating GWAS summary statistics from multiple ancestries and multiple CAD-related traits (Fig. 2). LDpred2 parameters selected for each score, whether the score survived after feature selection, and mixing weights from layers 1 and 2 are listed in Supplementary Table 1.

### GPS validation

The GPS_Mult_ was compared with previously published polygenic scores with respect to effect size for CAD association. The variant effect sizes were downloaded from PGS Catalog and calculated in the same UK Biobank validation dataset of 308,264 European ancestry individuals for direct comparison^8,9,23,28,49,68,69,84–96^. For score accession numbers and performance metrics, see Supplementary Table 3. The validation datasets were composed of UK Biobank participants separate from those used to train the GPS_Mult_. These individuals underwent genotyping using the UK BiLEVE Axiom Array or UK Biobank Axiom Array, containing over 800,000 variants spanning the genome^26^. Imputation was performed using the Haplotype Reference Consortium resource, the UK10K panel, and the 1000 Genomes panel^74,97,98^. We identified a subset of 488,243 participants with genotyping array data. After additional exclusion of 45,602 individuals for high heterozygosity or genotype missing rates, discordant reported versus genotypic sex, putative sex chromosome aneuploidy, excess relatedness (second-degree relative or closer), withdrawal of informed consent, or unreported ancestry and 116,649 individuals used for score training, 325,991 individuals (54.3% female, 2.2% African, 0.4% East Asian, 92.0% European and 2.7% South Asian) were included in the multi-ancestry internal validation cohort for subsequent analyses.

External validation was performed in the Million Veteran Program and Genes & Health studies. Among Million Veteran Program participants, 173,996 individuals not included in the previously published CAD GWAS^33^ were included and comprised 33,096 (21%) individuals of African ancestry and 124,467 (79%) individuals of European ancestry (Supplementary Table 2). Individuals were genotyped using the Affymetrix Axiom array and imputed to the TOPMed reference panel. Variants and sample quality control were previously described^99^. Within the Genes & Health study, individuals not included in the previously published CAD GWAS^34^ were included and comprised 16,874 participants of South Asian ancestry (Supplementary Table 2). These individuals underwent genotyping using the Illumina Infinium Global Screening Array v3 and imputed using the GenomeAsia pilot reference panel. Variants with low call rate (<0.99), rare variants with minor allele frequency <1%, and variants that failed the Hardy–Weinberg test (*P* < 1 × 10^−6^) in a subset of samples with low level of autozygosity were removed.

Across all cohorts, individuals were analyzed in distinct self-identified groups of African, East Asian, European, Hispanic and South Asian ancestries. The generated polygenic scores were residualized for the first ten principal components of genetic ancestry and then scaled to a mean of 0 and standard deviation of 1 for each ancestral group.

### Statistical analysis

Comparison of baseline characteristics between individuals with high or average genetic risk based on polygenic score was performed with the chi-squared test for categorical variables, analysis of variance (ANOVA) for a subset of continuous variables with normal distributions, and Mann–Whitney *U* test for continuous variables with nonparametric distributions. Individuals with a given magnitude of increased risk were identified by comparing progressively higher percentile cut-offs to the middle quintile population in a logistic regression model predicting disease status and adjusted for baseline model covariates. Individuals were next binned into 100 groupings according to percentile of the GPS_Mult_, and the unadjusted prevalence of CAD within each bin was determined.

Risk for prevalent disease was calculated using logistic regression models, including baseline model covariates defined as enrollment age, sex, genotyping array and the first ten principal components of genetic ancestry. Risk for incident CAD was calculated using Cox proportional-hazards regression models, including baseline model covariates. The proportion of phenotypic variance explained by the polygenic score or risk factor of interest on the observed scale was calculated using the Nagelkerke’s pseudo-*R*^2^ metric using the rcompanion R package—where *R*^2^ was calculated for the full model inclusive of the variable of interest plus the baseline model covariates minus *R*^2^ for the baseline model covariates alone. The proportion of phenotypic variance explained on the liability scale was similarly calculated using the logit liability *R*^2^ metric^100^.

To determine the polygenic risk equivalent of a CAD event comparable to risk experienced by those with prior CAD, a model was constructed comparing three groups and monitored for a CAD event in the follow-up period: individuals with prior CAD, individuals without prior CAD in different groupings of the top distribution of GPS_Mult_ (high GPS_Mult_) and individuals in the middle quintile of GPS_Mult_ without prior CAD using the survminer R package. Sequentially lower percentile cut-offs for this high GPS_Mult_ group were tested to find the grouping with equivalent risk increase for CAD as those with prior CAD. This analysis was repeated for diabetes mellitus, PAD and severe hypercholesterolemia (LDL cholesterol ≥190 mg/dL). In the lower tail of GPS_Mult_, the risk for incident CAD was calculated in individuals in the bottom 5 percentiles or 5th to 9th percentiles of GPS_Mult_ relative to those in the middle quintile, using Cox proportional-hazards regression models including baseline model covariates. The prevalence of CAD among individuals in the bottom 5 percentiles of GPS_Mult_ was calculated, stratified by 20 pack-years smoking increments and compared with the prevalence of CAD in nonsmokers in the middle 40th to 59th percentiles to estimate equivalent offset risk.

Cox proportional-hazards models were used to estimate HRs for incident CAD in the UK Biobank, with covariates of the first ten principal components. In model 1, only age and sex were modeled with the covariates. In model 2, only the clinical risk estimator—ACC/AHA PCE^5^ or QRISK3 (ref. ^6^)—was modeled with the covariates. In model 3, GPS_Mult_, clinical risk estimator, and the interaction term of GPS_Mult_ with the clinical risk estimator and the first ten principal components of genetic ancestry are modeled. The 10-year incidence of CAD for individuals grouped by GPS_Mult_ percentile and stratified by ancestry group was quantified using model 3 standardized to four PCE risk levels (mean 10-year risk of atherosclerotic cardiovascular disease as low (<5%), borderline (5% to <7.5%), intermediate (≥7.5% to <20%), and high (≥20%)) and the means of each of the covariates. The discrimination of each of these predictive models was assessed using Harrell’s C-statistic. The improvement in predictive performance of the addition of the GPS_Mult_ to the PCE or QRISK3 was evaluated using continuous and categorized NRI, with a risk probability threshold of 7.5% and 95% CIs obtained from 100-fold bootstrapping with the nricens R package. All analyses were two-sided. In all analyses, a 95% CI that excluded unity was considered evidence of statistical significance. All statistical analyses were performed with the use of R software, versions 3.5 and 3.6 (R Project for Statistical Computing) and figures were generated using the ggplot2 R package.

### Reporting summary

Further information on research design is available in the Nature Portfolio Reporting Summary linked to this article.

## Online content

Any methods, additional references, Nature Portfolio reporting summaries, source data, extended data, supplementary information, acknowledgements, peer review information; details of author contributions and competing interests; and statements of data and code availability are available at 10.1038/s41591-023-02429-x.

## Supplementary information

Supplementary Information Supplementary Tables 1–9.

Reporting Summary

## Data Availability

All data are made available from the UK Biobank (https://www.ukbiobank.ac.uk/enable-your-research/apply-for-access) to researchers from universities and other institutions with genuine research inquiries following institutional review board and UK Biobank approval. This research was conducted using the UK Biobank resource under application number 7089 and approved by the Mass General Brigham institutional review board. The genome-wide association data supporting the findings of this study are publicly available in Biobank Japan (http://jenger.riken.jp/en/result), FinnGen (https://www.finngen.fi/en/access_results), AGEN T2D (https://kp4cd.org/index.php/node/309), GIANT (https://portals.broadinstitute.org/collaboration/giant/), Global Lipids Genetics Consortium (http://csg.sph.umich.edu/willer/public/glgc-lipids2021) and Million Veteran Program (via dbGaP at https://ftp.ncbi.nlm.nih.gov/dbgap/studies/, under accession number phs001672), and upon request from CARDloGRAMplusC4D (http://www.cardiogramplusc4d.org/data-downloads/), MEGASTROKE (http://megastroke.org/download.html) and Genes & Health (https://www.genesandhealth.org/research/scientific-data-downloads). The full GPS_Mult_ weights are available in the Polygenic Score Catalog through accession ID PGS003725.
