# Supplementary material for: A multi-ancestry polygenic risk score improves risk prediction for coronary artery disease
Source: Nat Med. 2023 Jul 6;29(7):1793–803. doi: 10.1038/s41591-023-02429-x (PMC10353935; doi:10.1038/s41591-023-02429-x)
Supplement: Supplementary file 1 — Supplementary Tables 1–9. [file 41591_2023_2429_MOESM1_ESM.pdf]

nature medicine

Article

https://doi.org/10.1038/s41591-023-02429-x

A multi-ancestry polygenic risk score improves risk prediction for coronary artery disease

In the format provided by the authors and unedited

Table of Contents

Supplementary Tables:

Table 1: GPS_{Mult} inputs and parameters

Table 2: Summary of GPS_{Mult} training and validation datasets

Table 3: Performance of polygenic scores for CAD from Polygenic Score Catalog in the UK Biobank Study

Table 4: Baseline risk factor distributions and correlations with GPS_{Mult} in the UK Biobank Study

Table 5: Baseline characteristics according to high GPS_{Mult} in the UK Biobank Study

Table 6: Performance of polygenic scores for CAD from Polygenic Score Catalog in the Million Veteran Program Study

Table 7: Performance of polygenic scores for CAD from Polygenic Score Catalog in the Genes & Health Study

Table 8: Model C-statistics by ancestry in the UK Biobank Study

Table 9: Model net reclassification by ancestry in the UK Biobank Study

References

Consortia Acknowledgements

Table 1: GPS_{Mult} inputs and parameters

Trait	Name	Dominant Ancestry	N Cases	N Controls	ρ	h^2 scale	Is sparse LD	Train OR/SD	Layer 1 Mixing weight	Layer 2 Mixing weight	Final Weight	Reference
	CARDIO-GRAM plusC4D no UKBB	EUR	86847	417789	0.018	1	F	1.92	0.51		0.263	1
	BBJ	EAS	29319	183134	0.01	0.7	F	1.43	0.08		0.041	2
CAD	Genes & Health	SAS	1110	20898	0.0056	1.4	F	1.11	0.05	0.67	0.026	3
	FinnGen	EUR	33628	275526	0.0032	1.4	F	1.46	0.02		0.010	4
	MVP	EUR	95151	197287	0.018	0.7	F	1.72	0.23		0.120	5
	MVP	AFR	17202	59507	0.018	1.4	F	1.10	0.01		0.006	5
	MVP	HISP	6378	24270	0.0018	0.7	F	1.16	0.00		0.000	5
	GIANT	EUR	339224		1	1	T	1.12	0.11		0.014	6
BMI	BBJ	EAS	163835		0.32	0.7	F	1.06	0.05	0.03	0.007	2
	MVP	EUR	249262		0.032	1.4	F	1.11	0.12		0.038	7
DBP	BBJ	EAS	145515		0.01	0.7	F	1.07	0.06	0.08	0.019	2
	MVP	EUR	249262		0.032	0.7	F	1.19	0.19		0.000	7
SBP	BBJ	EAS	145505		0.018	1.4	F	1.09	0.05	0.00	0.000	2
DM	Diamante/ MVP	EUR	148726	965732	1	1.4	T	1.31	0.29	0.33	0.179	8

	MVP	AFR	24646	31446	0.018	0.7	F	1.06	0.00		0.000	[8]
	MVP	HISP	8,616	11,829	0.032	1.4	T	1.09	0.04		0.027	[8]
	AGEN T2D	EAS	77418	356122	0.56	1	T	1.14	0.01		0.005	[9]
	FinnGen	EUR	49303	255466	0.032	1.4	F	1.16	0.03		0.018	[4]
	Genes & Health	SAS	9044	12066	0.0056	1.4	F	1.05	0.00		0.000	[10]
	GLGC	EUR	1320016		0.1	0.7	T	1.22	0.26		0.028	[11]
	GLGC	AFR	99432		0.01	0.7	T	1.15	0.07		0.007	[11]
	GLGC	SAS	40963		0.00032	1.4	T	1.15	0.00		0.000	[11]
	MVP	EUR	215551		0.01	0.7	T	1.17	0.14		0.015	[12]
	MVP	AFR	57332		0.0056	0.7	T	1.13	0.05		0.005	[12]
	MVP	HISP	24742		0.0018	0.7	T	1.18	0.09		0.009	[12]
LDL-C	BBJ	EAS	72866		0.0032	0.7	T	1.13	0.00	0.09	0.000	[2]
	GLGC	EUR	1320016		1	0.7	T	1.24	0.07		0.009	[11]
	GLGC	AFR	99432		0.32	1.4	F	1.08	0.00		0.000	[11]
	GLGC	SAS	40963		0.001	1	T	1.05	0.04		0.006	[11]
	MVP	EUR	215551		0.32	1	F	1.20	0.20		0.026	[12]
HDL-C	MVP	AFR	57332		0.0056	1	F	1.07	0.00	0.06	0.000	[12]

	MVP	HISP	23946		0.056	1.4	F	1.08	0.00		0.000	12
	BBJ	EAS	74970		0.32	0.7	F	1.06	0.00		0.000	2
	GLGC	EUR	1320016		1	0.7	T	1.24	0.24		0.000	11
	GLGC	AFR	99432		0.018	1.4	F	1.09	0.00		0.000	11
	GLGC	SAS	40963		0.01	0.7	F	1.09	0.00		0.000	11
	MVP	EUR	215551		0.18	1	F	1.19	0.00		0.000	12
	MVP	AFR	57332		0.032	0.7	F	1.07	0.00		0.000	12
	MVP	HISP	24063		0.018	1.4	F	1.08	0.00		0.000	12
TG	BBJ	EAS	111667		0.056	0.7	T	1.07	0.00	0.00	0.000	2
	MVP	EUR	24009	150983	0.01	0.7	F	1.32	0.25		0.012	13
	MVP	AFR	5273	42485	1	1.4	T	1.07	0.05		0.002	13
	MVP	HISP	1925	18285	0.0032	1.4	T	1.07	0.03		0.002	13
	FinnGen	EUR	11924	288638	0.0056	0.7	F	1.16	0.08		0.004	4
PAD	BBJ	EAS	4112	173601	0.0032	0.7	F	1.13	0.09	0.04	0.004	2
	MEGA-STROKE	EUR	40585	406111	0.0032	0.7	T	1.17	0.17		0.030	14
	BBJ	EAS	22664	152022	0.56	1.4	T	1.06	0.05		0.010	2
Stroke	GBMI	AFR	1161	24416	0.018	1.4	T	1.04	0.05	0.07	0.010	15
CKD	MVP	EUR	223386		0.0001	0.7	T	1.05	0.04	0.06	0.025	16

	MVP	AFR	57336	0.018	1	F	1.04	0.03		0.019	[16]
	BBJ	EAS	150266	0.0056	0.7	T	1.02	0.00		0.000	[2]

Traits: CAD – coronary artery disease; BMI – body mass index; DBP – diastolic blood pressure; SBP – systolic blood pressure; DM – diabetes mellitus; LDL-C – low density lipoprotein cholesterol; HDL-C – high-density lipoprotein cholesterol; TG – triglycerides; PAD – peripheral artery disease; CKD – chronic kidney disease.

Consortia: CARDIOGRAMplusC4D no UKBB – Coronary ARtery DIsease Genome wide Replication and Meta-analysis plus The Coronary Artery Disease Genetics consortium excluding UK Biobank; BBJ – Biobank Japan; GIANT – Genetic Investigation of ANthropometric Traits; MVP – Million Veteran Program; AGEN-T2D – Asian Genetic Epidemiology Network Type 2 Diabetes Consortium; GLGC – Global Lipids Genetics Consortium excluding the UK Biobank; GBMI – Global Biobank Meta-analysis Initiative

Ancestries: AFR – African; EAS – East Asian; EUR – European; SAS – South Asian

LDPred2 parameters: ρ – tuning parameter to model the proportion of variants assumed to be causal; h^2 scale – factor by which heritability is scaled; is sparse LD – whether a sparse linkage disequilibrium matrix is applied, if true some variant effect estimates are fit to zero. A total of 102 combinations of these three parameters were possible and led to generation of 102 polygenic scores for each input ancestry-stratified set of GWAS summary statistics. The parameters leading to the best individually performing polygenic score in predicting CAD in a training dataset of 116,649 participants in the UK Biobank were chosen, specific to each ancestry-specific GWAS.

Mixing weights: Layer 1 – weight determined from a logistic regression model predicting CAD in a UK Biobank training dataset by combining different ancestry-specific GPS from GWAS for a specific trait. Layer 2 - weight determined from a logistic regression model predicting CAD in the same UKB training dataset by combining different multi-ancestry trait-specific scores from the Layer 1 step. Final weight - overall mixing weight incorporating proportional weights from layers 1 and 2, normalized to 100%. The final GPS_{Mult} score was a linear combination of all the input scores according to the final weight. The dataset for training the models and for estimating the mixing weight was from 116,649 participants in the UK Biobank. All the regression models for predicting CAD were adjusted for age, sex, genotyping array, and the first ten principal components of ancestry.

Table 2: Summary of GPS_{Mult} training and validation datasets

Stage	Training		Validation						
Cohort	UK Biobank	UK Biobank				MVP			G&H
Ancestry	European	African	East Asian	European	South Asian	African	European	Hispanic	South Asian
N	116649	7281	1464	308264	8982	33096	124467	16433	16874
Age (SD)	57.5 (7.9)	52.4 (8.0)	53.0 (7.6)	57.3 (8.0)	53.8 (8.5)	56.1 (12.4)	60.8 (13.3)	51.9 (15.5)	40.6 (14.5)
Male Sex (%)	47.5%	43.5%	37.2%	45.6%	54.1%	84.1%	91.3%	87.7%	45.9%
Case	4412	124	22	10492	542	4831	29171	2140	853
Control	112237	7157	1442	297772	8440	28265	95296	14293	16021

Baseline demographics of participants included in GPS_{Mult} training and validation. These individuals were not included in genome-wide association data used to construct GPS_{Mult}, and individuals in training and validation of GPS_{Mult} are distinct. MVP: Million Veteran Program; G&H: Genes & Health. SD: Standard deviation

Table 3: Performance of polygenic scores for CAD from Polygenic Score Catalog in the UK Biobank Study

Score Name	Identifier	Publication	OR/SD [95% CI]	P	Population
GRS28	PGS000200	Tikkanen E et al. Arterioscler Thromb Vasc Biol (2013)	1.31 [1.31-1.34]	$<2x10^{-16}$	EUR
GRS27	PGS000010	Mega JL et al. Lancet (2015)	1.35 [1.35-1.38]	$<2x10^{-16}$	EUR
GRS50	PGS000011	Tada H et al. Eur Heart J (2015)	1.34 [1.34-1.37]	$<2x10^{-16}$	EUR
GRS49K	PGS000012	Abraham G et al. Eur Heart J (2016)	1.44 [1.44-1.47]	$<2x10^{-16}$	EUR
CHD57	PGS000057	Natarajan P et al. Circulation (2017)	1.34 [1.34-1.37]	$<2x10^{-16}$	EUR
GRS_CAD	PGS000019	Paquette M et al. J Clin Lipidol (2017)	1.49 [1.49-1.52]	$<2x10^{-16}$	EUR
GPS_{2018}	PGS000013	Khera AV et al. Nat Genet (2018)	1.72 [1.72-1.76]	$<2x10^{-16}$	EUR
metaGRS_CAD	PGS000018	Inouye M et al. J Am Coll Cardiol (2018)	1.77 [1.77-1.8]	$<2x10^{-16}$	EUR
CHD46	PGS000059	Hajek C et al. Circ Genom Precis Med (2018)	1.27 [1.27-1.3]	$<2x10^{-16}$	EUR
CAD_EJ2020	PGS000116	Elliott J et al. JAMA (2020)	1.53 [1.53-1.56]	$<2x10^{-16}$	EUR
PRS_COMBINED	PGS000749	Gola D et al. Circ Genom Precis Med (2020)	1.49 [1.49-1.52]	$<2x10^{-16}$	EUR
PRS_CHD	PGS000329	Mars N et al. Nat Med (2020)	1.64 [1.64-1.68]	$<2x10^{-16}$	EUR
GPS_CAD_SA	PGS000296	Wang M et al. J Am Coll Cardiol (2020)	1.67 [1.67-1.71]	$<2x10^{-16}$	EUR
PRS70_CAD	PGS000349	Pechlivanis S et al. BMC Med Genet (2020)	1.14 [1.14-1.17]	$<2x10^{-16}$	EUR
GRS_Metabo	PGS000818	Bauer A et al. Genet Epidemiol (2021)	1.48 [1.48-1.51]	$<2x10^{-16}$	EUR
$AnnoPred_{CAD}$	PGS001355	Ye Y et al. Circ Genom Precis Med (2021)	1.76 [1.76-1.8]	$<2x10^{-16}$	EUR
metaPRS_CAD	PGS002262	Lu X et al. Eur Heart J (2022)	1.30 [1.3-1.32]	$<2x10^{-16}$	EUR

CHD_PRSCS	PGS001780	Tamlander M et al. Commun Biol (2022)	1.73 [1.73-1.77]	$<2x10^{-16}$	EUR
GPS_{Mult}	-	-	2.14 [2.10-2.19]	$<2x10^{-16}$	EUR

A series of prior published scores or CAD from the Polygenic Score Catalog that did not include any UK Biobank participants in the GWAS data for score development were compared with GPS_{Mult} within the validation cohort of European ancestry individuals from the UK Biobank. The odds ratio for prevalent coronary artery disease (CAD) risk per standard deviation (ORS/SD) of the polygenic score and 95% confidence interval were assessed in a logistic regression model adjusted for age, sex, genotyping array, and the first ten principal components of ancestry. *P* values are derived from a *t*-test implemented in the GLM function in R and are two-sided. Beta: Effect size from logistic regression; SE: standard error.

Table 4: Baseline risk factor distributions and correlations with GPS_{Mult}

	Prevalent CAD	Incident CAD	No CAD	All	Correlation
**N**	11180	11898	302913	325991	
**LDL Cholesterol* (mg/dl, mean (SD))**	143.78 (35.76)	156.08 (35.81)	145.25 (32.95)	145.60 (33.22)	0.152
**HDL Cholesterol* (mg/dl, mean (SD))**	46.20 (12.16)	50.49 (13.19)	56.72 (14.81)	56.13 (14.84)	-0.115
**Triglycerides* (mg/dl, median [IQR])**	170.17 [118.87, 243.84]	167.25 [117.68, 241.03]	131.98 [92.65, 192.47]	134.37 [93.98, 196.19]	0.108
**Systolic Blood Pressure (mm Hg, mean (SD))**	140.00 (19.86)	147.28 (20.06)	139.37 (19.56)	139.68 (19.64)	0.081
**Diastolic Blood Pressure (mm Hg, mean (SD))**	78.94 (10.93)	84.57 (10.94)	82.27 (10.66)	82.24 (10.71)	0.071
**Body-mass Index (kg/m^2, mean (SD))**	29.13 (4.77)	28.58 (4.79)	27.26 (4.74)	27.37 (4.76)	0.108
**Diabetes Mellitus# (%)**	2197 (19.7)	1460 (12.3)	14012 (4.6)	17669 (5.4)	0.028
**Chronic Kidney Disease# (%)**	1621 (15.2)	1134 (10.0)	11484 (4.0)	14239 (4.6)	0.091

The baseline variable distributions for traditional coronary artery disease (CAD) risk factors stratified by CAD case status. Correlations between the GPS_{Mult} and risk factors across the population, adjusted for top 10 principal components and traditional risk factors were assessed using Pearson's correlation for continuous traits and #point biserial correlation for binary traits. The P value for each association was less than 2×10^{-12}. *LDL-C, HDL-C, and triglyceride values adjusted if individual taking cholesterol lowering medications as described in Patel et al. *JAMA Network Open* 2020.[17] For example, in the case of statin intake, lower density cholesterol was divided by 0.7 and triglycerides by 0.85. Diagnosis of diabetes was based on self-report, primary care records or hospitalization records confirming a clinical diagnosis, self-reported consumption of medications to treat diabetes, or glycohemoglobin >6.5% at enrollment and chronic kidney disease was defined as eGFRcys <60 mL/min, as previously described.[18]

Table 5: Baseline characteristics according to high GPS_{Mult}

	Bottom 80% GPS_{Mult}	Top 20% GPS_{Mult}
Number of individuals	224,292	56,424
Prevalent coronary artery disease (%)	5,496 (2.5)	4,155 (7.4)
Age, years (mean, SD)	57.5 (7.9)	57.2 (9.0)
Male sex (%)	102,639 (45.8)	25,926 (45.9)
Hypertension (%)	58,635 (27.2)	20,305 (37.9)
Diabetes Mellitus (%)	9,219 (4.1)	4,653 (8.2)
*LDL cholesterol, mg/dL (mean (SD))	137.7 (33.0)	140.5 (35.9)
*HDL cholesterol, mg/dL (mean (SD))	57.0 (14.9)	53.9 (14.3)
*Triglycerides, mg/dL (median [IQR])	129.4 [91.6, 186.8]	142.3 [100.7, 204.9]
Current smoking (%)	20,365 (9.1)	5,866 (10.4)
Family history of heart disease (%)	95,049 (42.4)	29,936 (53.1)
Body mass index, kg/m^2 (mean (SD))	27.2 (4.7)	28.1 (5.0)
Lipid-lowering therapy (%)	31,201 (13.9)	13,892 (24.6)

Baseline characteristics according to high GPS_{Mult} status, defined as the top 20% of the distribution empirically shown to be at ≥3-fold risk of coronary artery disease. LDL: low-density lipoprotein; HDL: high-density lipoprotein. *LDL-C, HDL-C, and triglyceride values were adjusted for cholesterol-lowering medication status, as previously described.[17]

Table 6: Performance of polygenic scores for CAD from Polygenic Score Catalog in the Million Veteran Program Study

Score Name	Identifier	Publication	Beta	SE	P	OR/SD [95% CI]	Population
GRS28	PGS000200	Tikkanen E et al. Arterioscler Thromb Vasc Biol (2013)	0.053	0.016	1.20E-03	1.05 (1.02-1.09)	AFR
GRS27	PGS000010	Mega JL et al. Lancet (2015)	0.066	0.016	4.60E-05	1.07 (1.04-1.1)	AFR
GRS50	PGS000011	Tada H et al. Eur Heart J (2015)	0.073	0.016	6.30E-06	1.08 (1.04-1.11)	AFR
GRS49K	PGS000012	Abraham G et al. Eur Heart J (2016)	0.082	0.016	4.70E-07	1.09 (1.05-1.12)	AFR
CHD57	PGS000057	Natarajan P et al. Circulation (2017)	0.066	0.016	6.20E-05	1.07 (1.03-1.1)	AFR
GRS_CAD	PGS000019	Paquette M et al. J Clin Lipidol (2017)	0.115	0.016	2.60E-12	1.12 (1.09-1.16)	AFR
GPS_{2018}	PGS000013	Khera AV et al. Nat Genet (2018)	0.128	0.016	3.10E-15	1.14 (1.1-1.17)	AFR
CAD_GRS_204	PGS000058	Morieri ML et al. Diabetes Care (2018)	0.117	0.016	1.10E-12	1.12 (1.09-1.16)	AFR
metaGRS_CAD	PGS000018	Inouye M et al. J Am Coll Cardiol (2018)	0.155	0.016	2.60E-21	1.17 (1.13-1.21)	AFR
CHD46	PGS000059	Hajek C et al. Circ Genom Precis Med (2018)	0.060	0.016	2.20E-04	1.06 (1.03-1.1)	AFR
157SNP_GRS	PGS000798	Severance LM et al. J Cardiovasc Comput Tomogr (2019)	0.086	0.016	1.30E-07	1.09 (1.06-1.13)	AFR
CAD_EJ2020	PGS000116	Elliott J et al. JAMA (2020)	0.114	0.016	4.30E-12	1.12 (1.08-1.16)	AFR
PRS_DE	PGS000748	Gola D et al. Circ Genom Precis Med (2020)	0.118	0.016	3.20E-13	1.13 (1.09-1.16)	AFR
PRS_COMBINED	PGS000749	Gola D et al. Circ Genom Precis Med (2020)	0.112	0.016	4.50E-12	1.12 (1.08-1.16)	AFR
PRS_CHD	PGS000329	Mars N et al. Nat Med (2020)	0.144	0.016	1.00E-18	1.15 (1.12-1.19)	AFR
GPS_CAD_SA	PGS000296	Wang M et al. J Am Coll Cardiol (2020)	0.158	0.016	2.50E-22	1.17 (1.13-1.21)	AFR
PRS70_CAD	PGS000349	Pechlivanis S et al. BMC Med Genet (2020)	0.028	0.016	8.10E-02	1.03 (1-1.06)	AFR
$MetaPRS_{CAD}$	PGS000337	Koyama S et al. Nat Genet (2020)	0.167	0.016	3.00E-24	1.18 (1.14-1.22)	AFR
PRS176_CHD	PGS000899	Feitosa MF et al. Circ Genom Precis Med (2021)	0.109	0.016	3.40E-11	1.11 (1.08-1.15)	AFR

GRS_Metabo	PGS000818	Bauer A et al. Genet Epidemiol (2021)	0.101	0.016	6.80E-10	1.11 (1.07-1.14)	AFR
AnnoPred$_{CAD}$	PGS001355	Ye Y et al. Circ Genom Precis Med (2021)	0.161	0.016	5.10E-23	1.18 (1.14-1.21)	AFR
portability-ldpred2	PGS002048	Prive F et al. Am J Hum Genet (2022)	0.129	0.016	5.30E-15	1.14 (1.1-1.17)	AFR
metaPRS_CAD	PGS002262	Lu X et al. Eur Heart J (2022)	0.061	0.016	2.10E-04	1.06 (1.03-1.1)	AFR
PRSCS$_{CHD}$	PGS001780	Tamlander M et al. Commun Biol (2022)	0.166	0.016	5.40E-24	1.18 (1.14-1.22)	AFR
GBE_HC942	PGS000962	Tanigawa Y et al. PLoS Genet (2022)	0.088	0.016	6.60E-08	1.09 (1.06-1.13)	AFR
ldpred_cad	PGS002244	Mars N et al. Cell Genom (2022)	0.139	0.016	1.70E-17	1.15 (1.11-1.19)	AFR
PRS_2022	PGS003356	Aragam KA et al. Nat Genet (2022)	0.160	0.016	1.10E-22	1.17 (1.14-1.21)	AFR
GPSMult	-	-	0.222	0.016	2.20E-41	1.25 (1.21-1.29)	AFR
GRS28	PGS000200	Tikkanen E et al. Arterioscler Thromb Vasc Biol (2013)	0.185	0.007	7.50E-148	1.2 (1.19-1.22)	EUR
GRS27	PGS000010	Mega JL et al. Lancet (2015)	0.214	0.007	4.40E-199	1.24 (1.22-1.26)	EUR
GRS50	PGS000011	Tada H et al. Eur Heart J (2015)	0.195	0.007	5.80E-163	1.21 (1.2-1.23)	EUR
GRS49K	PGS000012	Abraham G et al. Eur Heart J (2016)	0.249	0.007	4.80E-262	1.28 (1.27-1.3)	EUR
CHD57	PGS000057	Natarajan P et al. Circulation (2017)	0.118	0.007	8.80E-62	1.13 (1.11-1.14)	EUR
GRS_CAD	PGS000019	Paquette M et al. J Clin Lipidol (2017)	0.273	0.007	1.1E-312	1.31 (1.3-1.33)	EUR
GPS_CAD	PGS000013	Khera AV et al. Nat Genet (2018)	0.370	0.007	5.6E-551	1.45 (1.43-1.47)	EUR
CAD_GRS_204	PGS000058	Morieri ML et al. Diabetes Care (2018)	0.326	0.007	8.4E-439	1.39 (1.37-1.41)	EUR
metaGRS_CAD	PGS000018	Inouye M et al. J Am Coll Cardiol (2018)	0.385	0.007	1.2E-593	1.47 (1.45-1.49)	EUR
CHD46	PGS000059	Hajek C et al. Circ Genom Precis Med (2018)	0.165	0.007	1.00E-118	1.18 (1.16-1.2)	EUR
157SNP_GRS	PGS000798	Severance LM et al. J Cardiovasc Comput Tomogr (2019)	0.289	0.007	4.0E-347	1.33 (1.32-1.35)	EUR
CAD_EJ2020	PGS000116	Elliott J et al. JAMA (2020)	0.295	0.007	6.0E-359	1.34 (1.32-1.36)	EUR
PRS_DE	PGS000748	Gola D et al. Circ Genom Precis Med (2020)	0.273	0.007	3.1E-312	1.31 (1.3-1.33)	EUR

PRS_COMBINED	PGS000749	Gola D et al. Circ Genom Precis Med (2020)	0.275	0.007	2.9E-316	1.32 (1.3-1.33)	EUR
PRS_CHD	PGS000329	Mars N et al. Nat Med (2020)	0.367	0.007	1.5E-551	1.44 (1.42-1.46)	EUR
GPS_CAD_SA	PGS000296	Wang M et al. J Am Coll Cardiol (2020)	0.356	0.007	2.7E-515	1.43 (1.41-1.45)	EUR
PRS70_CAD	PGS000349	Pechlivanis S et al. BMC Med Genet (2020)	0.104	0.007	1.30E-48	1.11 (1.09-1.13)	EUR
$MetaPRS_{CAD}$	PGS000337	Koyama S et al. Nat Genet (2020)	0.418	0.007	1.5E-688	1.52 (1.5-1.54)	EUR
PRS176_CHD	PGS000899	Feitosa MF et al. Circ Genom Precis Med (2021)	0.327	0.007	2.8E-441	1.39 (1.37-1.41)	EUR
GRS_Metabo	PGS000818	Bauer A et al. Genet Epidemiol (2021)	0.258	0.007	1.80E-279	1.29 (1.28-1.31)	EUR
$AnnoPred_{CAD}$	PGS001355	Ye Y et al. Circ Genom Precis Med (2021)	0.383	0.007	7.3E-585	1.47 (1.45-1.49)	EUR
portability-ldpred2	PGS002048	Prive F et al. Am J Hum Genet (2022)	0.379	0.007	2.0E-580	1.46 (1.44-1.48)	EUR
metaPRS_CAD	PGS002262	Lu X et al. Eur Heart J (2022)	0.207	0.007	2.00E-182	1.23 (1.21-1.25)	EUR
$PRSCS_{CHD}$	PGS001780	Tamlander M et al. Commun Biol (2022)	0.372	0.007	3.9E-556	1.45 (1.43-1.47)	EUR
GBE_HC942	PGS000962	Tanigawa Y et al. PLoS Genet (2022)	0.287	0.007	6.2E-345	1.33 (1.31-1.35)	EUR
ldpred_cad	PGS002244	Mars N et al. Cell Genom (2022)	0.344	0.007	4.3E-482	1.41 (1.39-1.43)	EUR
PRS_{2022}	PGS003356	Aragam KA et al. Nat Genet (2022)	0.477	0.008	1.4E-874	1.61 (1.59-1.64)	EUR
GPS_{Mult}	-	-	0.542	0.008	1.1E-1085	1.72 (1.69-1.75)	EUR
GRS28	PGS000200	Tikkanen E et al. Arterioscler Thromb Vasc Biol (2013)	0.123	0.025	1.10E-06	1.13 (1.08-1.19)	HIS
GRS27	PGS000010	Mega JL et al. Lancet (2015)	0.147	0.025	5.70E-09	1.16 (1.1-1.22)	HIS
GRS50	PGS000011	Tada H et al. Eur Heart J (2015)	0.136	0.025	8.20E-08	1.15 (1.09-1.2)	HIS
GRS49K	PGS000012	Abraham G et al. Eur Heart J (2016)	0.217	0.025	1.40E-17	1.24 (1.18-1.31)	HIS
CHD57	PGS000057	Natarajan P et al. Circulation (2017)	0.127	0.025	5.10E-07	1.14 (1.08-1.19)	HIS
GRS_CAD	PGS000019	Paquette M et al. J Clin Lipidol (2017)	0.236	0.025	9.70E-21	1.27 (1.2-1.33)	HIS
GPS_{2018}	PGS000013	Khera AV et al. Nat Genet (2018)	0.286	0.026	9.50E-29	1.33 (1.27-1.4)	HIS

CAD_GRS_204	PGS000058	Morieri ML et al. Diabetes Care (2018)	0.286	0.026	6.60E-29	1.33 (1.27-1.4)	HIS
metaGRS_CAD	PGS000018	Inouye M et al. J Am Coll Cardiol (2018)	0.320	0.025	2.90E-36	1.38 (1.31-1.45)	HIS
CHD46	PGS000059	Hajek C et al. Circ Genom Precis Med (2018)	0.159	0.025	3.50E-10	1.17 (1.12-1.23)	HIS
157SNP_GRS	PGS000798	Severance LM et al. J Cardiovasc Comput Tomogr (2019)	0.244	0.025	9.10E-22	1.28 (1.21-1.34)	HIS
CAD_EJ2020	PGS000116	Elliott J et al. JAMA (2020)	0.251	0.026	1.10E-22	1.29 (1.22-1.35)	HIS
PRS_DE	PGS000748	Gola D et al. Circ Genom Precis Med (2020)	0.254	0.025	9.80E-24	1.29 (1.23-1.36)	HIS
PRS_COMBINED	PGS000749	Gola D et al. Circ Genom Precis Med (2020)	0.238	0.025	4.40E-21	1.27 (1.21-1.33)	HIS
PRS_CHD	PGS000329	Mars N et al. Nat Med (2020)	0.285	0.025	4.50E-29	1.33 (1.27-1.4)	HIS
GPS_CAD_SA	PGS000296	Wang M et al. J Am Coll Cardiol (2020)	0.291	0.026	4.50E-30	1.34 (1.27-1.41)	HIS
PRS70_CAD	PGS000349	Pechlivanis S et al. BMC Med Genet (2020)	0.091	0.025	2.90E-04	1.1 (1.04-1.15)	HIS
$MetaPRS_{CAD}$	PGS000337	Koyama S et al. Nat Genet (2020)	0.398	0.026	2.30E-52	1.49 (1.41-1.57)	HIS
PRS176_CHD	PGS000899	Feitosa MF et al. Circ Genom Precis Med (2021)	0.280	0.026	6.30E-28	1.32 (1.26-1.39)	HIS
GRS_Metabo	PGS000818	Bauer A et al. Genet Epidemiol (2021)	0.218	0.025	1.40E-17	1.24 (1.18-1.31)	HIS
$AnnoPred_{CAD}$	PGS001355	Ye Y et al. Circ Genom Precis Med (2021)	0.349	0.026	1.10E-41	1.42 (1.35-1.49)	HIS
portability-ldpred2	PGS002048	Prive F et al. Am J Hum Genet (2022)	0.337	0.026	2.40E-39	1.4 (1.33-1.47)	HIS
metaPRS_CAD	PGS002262	Lu X et al. Eur Heart J (2022)	0.202	0.025	2.60E-15	1.22 (1.16-1.29)	HIS
$PRSCS_{CHD}$	PGS001780	Tamlander M et al. Commun Biol (2022)	0.330	0.026	1.50E-37	1.39 (1.32-1.46)	HIS
GBE_HC942	PGS000962	Tanigawa Y et al. PLoS Genet (2022)	0.222	0.025	2.30E-18	1.25 (1.19-1.31)	HIS
ldpred_cad	PGS002244	Mars N et al. Cell Genom (2022)	0.269	0.026	4.60E-26	1.31 (1.25-1.38)	HIS
PRS_2022	PGS003356	Aragam KA et al. Nat Genet (2022)	0.418	0.026	1.40E-57	1.52 (1.44-1.6)	HIS
GPSMult	-	-	0.477	0.027	5.10E-72	1.61 (1.53-1.7)	HIS

The odds ratio for prevalent coronary artery disease (CAD) risk per standard deviation (OR/SD) of the polygenic score and 95% confidence interval were validated in a logistic regression model adjusted for age, sex, genotyping array, and the first ten principal components of ancestry

in a holdout cohort of individuals of the Million Veteran Project using prior published scores from the Polygenic Score Catalog. *P* values are derived from a *t*-test implemented in the GLM function in R and are two-sided. Beta: Effect size from logistic regression; SE: standard error. AFR: African ancestry; EUR: European ancestry; HISP: Hispanic ancestry.

Table 7: Performance of polygenic scores for CAD from Polygenic Score Catalog in the Genes & Health Study

Score Name	Identifier	Publication	Beta	SE	P	OR/SD [95% CI]	Population
GRS28	PGS000200	Tikkanen E et al. Arterioscler Thromb Vasc Biol (2013)	0.169	0.040	2.40E-05	1.18 [1.09-1.28]	SAS
GRS27	PGS000010	Mega JL et al. Lancet (2015)	0.159	0.040	6.79E-05	1.17 [1.08-1.27]	SAS
GRS50	PGS000011	Tada H et al. Eur Heart J (2015)	0.108	0.039	5.91E-03	1.11 [1.03-1.2]	SAS
GRS49K	PGS000012	Abraham G et al. Eur Heart J (2016)	0.288	0.044	4.38E-11	1.33 [1.22-1.45]	SAS
CHD57	PGS000057	Natarajan P et al. Circulation (2017)	0.105	0.040	9.17E-03	1.11 [1.03-1.2]	SAS
GRS_CAD	PGS000019	Paquette M et al. J Clin Lipidol (2017)	0.218	0.040	4.99E-08	1.24 [1.15-1.34]	SAS
GPS_{2018}	PGS000013	Khera AV et al. Nat Genet (2018)	0.285	0.042	7.74E-12	1.33 [1.23-1.44]	SAS
CAD_GRS_204	PGS000058	Morieri ML et al. Diabetes Care (2018)	0.229	0.040	1.23E-08	1.26 [1.16-1.36]	SAS
metaGRS_CAD	PGS000018	Inouye M et al. J Am Coll Cardiol (2018)	0.327	0.044	9.81E-14	1.39 [1.27-1.51]	SAS
CHD46	PGS000059	Hajek C et al. Circ Genom Precis Med (2018)	0.111	0.040	5.83E-03	1.12 [1.03-1.21]	SAS
157SNP_GRS	PGS000798	Severance LM et al. J Cardiovasc Comput Tomogr (2019)	0.244	0.041	2.07E-09	1.28 [1.18-1.38]	SAS
CAD_EJ2020	PGS000116	Elliott J et al. JAMA (2020)	0.327	0.042	4.18E-15	1.39 [1.28-1.5]	SAS
PRS_COMBINED	PGS000749	Gola D et al. Circ Genom Precis Med (2020)	0.328	0.041	2.26E-15	1.39 [1.28-1.51]	SAS
PRS_DE	PGS000748	Gola D et al. Circ Genom Precis Med (2020)	0.306	0.041	1.13E-13	1.36 [1.25-1.47]	SAS
PRS_CHD	PGS000329	Mars N et al. Nat Med (2020)	0.310	0.041	2.26E-14	1.36 [1.26-1.48]	SAS
GPS_CAD_SA	PGS000296	Wang M et al. J Am Coll Cardiol (2020)	0.338	0.041	3.57E-16	1.4 [1.29-1.52]	SAS
PRS70_CAD	PGS000349	Pechlivanis S et al. BMC Med Genet (2020)	0.072	0.040	6.87E-02	1.08 [0.99-1.16]	SAS
$MetaPRS_{CAD}$	PGS000337	Koyama S et al. Nat Genet (2020)	0.428	0.042	1.17E-24	1.53 [1.41-1.67]	SAS
PRS176_CHD	PGS000899	Feitosa MF et al. Circ Genom Precis Med (2021)	0.251	0.041	5.86E-10	1.29 [1.19-1.39]	SAS
GRS_Metabo	PGS000818	Bauer A et al. Genet Epidemiol (2021)	0.209	0.040	2.13E-07	1.23 [1.14-1.33]	SAS

CAD_AnnoPred	PGS001355	Ye Y et al. Circ Genom Precis Med (2021)	0.352	0.041	1.71E-17	1.42 [1.31-1.54]	SAS
portability-ldpred2	PGS002048	Priv√© F et al. Am J Hum Genet (2022)	0.380	0.041	4.53E-20	1.46 [1.35-1.59]	SAS
metaPRS_CAD	PGS002262	Lu X et al. Eur Heart J (2022)	0.193	0.041	6.24E-06	1.21 [1.12-1.31]	SAS
$PRSCS_{CHD}$	PGS001780	Tamlander M et al. Commun Biol (2022)	0.420	0.042	3.64E-23	1.52 [1.4-1.65]	SAS
GBE_HC942	PGS000962	Tanigawa Y et al. PLoS Genet (2022)	0.180	0.040	2.69E-06	1.2 [1.11-1.29]	SAS
ldpred_cad	PGS002244	Mars N et al. Cell Genom (2022)	0.301	0.042	4.13E-13	1.35 [1.25-1.47]	SAS
PRS_{2022}	PGS003356	Aragam KA et al. Nat Genet (2022)	0.469	0.042	1.37E-29	1.6 [1.47-1.73]	SAS
GPS_{Mult}	-	-	0.606	0.042	1.95E-47	1.83 [1.69-1.99]	SAS

The odds ratio for prevalent coronary artery disease (CAD) risk per standard deviation (OR/SD) of the polygenic score and 95% confidence interval were validated in a logistic regression model adjusted for age, sex, genotyping array, and the first ten principal components of ancestry in a holdout cohort of individuals of the Genes & Health study using prior published scores from the Polygenic Score Catalog. *P* values are derived from a *t*-test implemented in the GLM function in R and are two-sided. Beta: Effect size from logistic regression; SE: standard error. SAS: South Asian ancestry.

Table 8: Model C-statistics by ancestry in the UK Biobank Study

Ancestry	All	African	East Asian	European	South Asian
**Age+Sex**	0.71 (0.706-0.715)	0.708 (0.666-0.75)	0.775 (0.694-0.856)	0.708 (0.703-0.712)	0.712 (0.692-0.732)
**PCE**	0.739 (0.735-0.744)	0.749 (0.707-0.791)	0.803 (0.71-0.895)	0.739 (0.734-0.743)	0.739 (0.717-0.76)
**PCE+GPS_{Mult}+PCE*GPS_{Mult}**	0.763 (0.759-0.768)	0.753 (0.712-0.794)	0.882 (0.843-0.921)	0.762 (0.758-0.767)	0.764 (0.743-0.784)
**QRISK3**	0.746 (0.741-0.75)	0.747 (0.698-0.796)	0.811 (0.73-0.893)	0.744 (0.74-0.749)	0.745 (0.722-0.769)
**QRISK3+GPS_{Mult}+QRISK3*GPS_{Mult}**	0.769 (0.764-0.773)	0.759 (0.712-0.806)	0.882 (0.834-0.929)	0.768 (0.763-0.772)	0.777 (0.756-0.799)

C-statistics stratified by ancestry are based on 10-year follow-up events from Cox regression models of listed variables, the first ten principal components of genetic ancestry, and genotyping array. ACC/AHA Pooled Cohort Equations (PCE) and QISK3 10-year risk estimates include age and sex variables in their risk estimation.

Table 9: Model net reclassification by ancestry in the UK Biobank Study

NRI		Status	All	African	East Asian	European	South Asian
Categorical	PCE	Full	0.070 (0.059-0.082)	0.010 (-0.016-0.035)	0.000 (-0.008-0.176)	0.073 (0.063-0.081)	0.067 (0.019-0.147)
		Cases	0.081 (0.069-0.094)	0.010 (-0.016-0.034)	0.000 (0.000-0.179)	0.083 (0.074-0.092)	0.102 (0.043-0.188)
		Noncases	-0.011 (-0.012 - -0.009)	0.00 (-0.003-0.002)	0000 (-0.008-0.003)	-0.011 (-0.012-0.009)	-0.035 (-0.053-0.018)
	QRISK3	Full	0.034 (0.028-0.040)	0.011 (-0.014-0.058)	-0.001 (-0.003-0.002)	0.036 (0.029-0.044)	0.025 (-0.011-0.101)
		Cases	0.038 (0.032-0.045)	0.012 (-0.012-0.059)	0.000 (0.000-0.000)	0.040 (0.033-0.049)	0.035 (-0.004-0.124)
		Noncases	-0.004 (-0.005 - -0.003)	-0.001 (-0.003-0.001)	-0.001 (-0.003-0.002)	-0.005 (-0.006-0.004)	-0.011 (-0.023-0.003)
Continuous	PCE	Full	0.405 (0.379-0.424)	0.057 (-0.211-0.291)	0.427 (-0.249-0.849)	0.412 (0.388-0.431)	0.366 (0.269-0.463)
		Cases	0.200 (0.182-0.215)	-0.026 (-0.245-0.184)	0.285 (-0.402-0.599)	0.206 (0.186-0.221)	0.175 (0.104-0.257)
		Noncases	0.205 (0.197-0.213)	0.084 (-0.012-0.183)	0.142 (-0.007-0.347)	0.207 (0.195-0.217)	0.191 (0.158-0.233)
	QRISK3	Full	0.395 (0.373-0.418)	0.173 (-0.207-0.673)	0.300 (-0.338-0.780)	0.403 (0.377-0.426)	0.355 (0.232-0.456)
		Cases	0.195 (0.177-0.214)	0.059 (-0.165-0.443)	0.164 (-0.496-0.594)	0.201 (0.183-0.218)	0.173 (0.073-0.246)
		Noncases	0.200 (0.192-0.210)	0.114 (-0.013-0.406)	0.137 (-0.015-0.383)	0.202 (0.193-0.210)	0.183 (0.138-0.243)

The improvement in the predictive performance of the addition of the GPS_{Mult} to the ACC/AHA Pooled Cohort Equations (PCE) or QRISK3 was evaluated in each ancestry using continuous and categorised net reclassification improvement (NRI), with a risk probabilities threshold of 7.5% obtained with Kaplan-Meier estimates for a period of 10 years and confidence intervals (95%) obtained from 100-fold bootstrapping.

References:

1. Aragam, K. G. *et al.* Discovery and systematic characterization of risk variants and genes for coronary artery disease in over a million participants. 2021.05.24.21257377 Preprint at https://doi.org/10.1101/2021.05.24.21257377 (2021).
2. Sakaue, S. *et al.* A cross-population atlas of genetic associations for 220 human phenotypes. *Nat Genet* **53**, 1415–1424 (2021).
3. Huang, Q. Q. *et al.* Transferability of genetic loci and polygenic scores for cardiometabolic traits in British Pakistani and Bangladeshi individuals. *Nat Commun* **13**, 4664 (2022).
4. Kurki, M. I. *et al.* FinnGen: Unique genetic insights from combining isolated population and national health register data. 2022.03.03.22271360 Preprint at https://doi.org/10.1101/2022.03.03.22271360 (2022).
5. Tcheandjieu, C. *et al.* Large-scale genome-wide association study of coronary artery disease in genetically diverse populations. *Nat Med* 1–13 (2022) doi:10.1038/s41591-022-01891-3.
6. Locke, A. E. *et al.* Genetic studies of body mass index yield new insights for obesity biology. *Nature* **518**, 197–206 (2015).
7. Evangelou, E. *et al.* Genetic analysis of over 1 million people identifies 535 new loci associated with blood pressure traits. *Nat Genet* **50**, 1412–1425 (2018).
8. Vujkovic, M. *et al.* Discovery of 318 new risk loci for type 2 diabetes and related vascular outcomes among 1.4 million participants in a multi-ancestry meta-analysis. *Nat Genet* **52**, 680–691 (2020).
9. Spracklen, C. N. *et al.* Identification of type 2 diabetes loci in 433,540 East Asian individuals. *Nature* **582**, 240–245 (2020).
10. Hodgson, S. *et al.* Harnessing the power of polygenic risk scores to predict type 2 diabetes and its subtypes in a high-risk population of British Pakistanis and Bangladeshis in a routine healthcare setting. 2021.07.12.21259837 Preprint at https://doi.org/10.1101/2021.07.12.21259837 (2021).
11. Graham, S. E. *et al.* The power of genetic diversity in genome-wide association studies of lipids. *Nature* **600**, 675–679 (2021).
12. Klarin, D. *et al.* Genetics of blood lipids among ~300,000 multi-ethnic participants of the Million Veteran Program. *Nat Genet* **50**, 1514–1523 (2018).
13. Klarin, D. *et al.* Genome-wide Association Study of Peripheral Artery Disease in the Million Veteran Program. *Nat Med* **25**, 1274–1279 (2019).
14. Malik, R. *et al.* Multiancestry genome-wide association study of 520,000 subjects identifies 32 loci associated with stroke and stroke subtypes. *Nat Genet* **50**, 524–537 (2018).
15. Zhou, W. *et al.* Global Biobank Meta-analysis Initiative: powering genetic discovery across human diseases. 2021.11.19.21266436 Preprint at https://doi.org/10.1101/2021.11.19.21266436 (2021).
16. Hellwege, JN *et al*. Mapping eGFR loci to the renal transcriptome and phenome in the VA Million Veteran Program. *Nat Commun.* **10**(1):3842 (2019).
17. Patel, A. P. *et al.* Association of Rare Pathogenic DNA Variants for Familial Hypercholesterolemia, Hereditary Breast and Ovarian Cancer Syndrome, and Lynch Syndrome With Disease Risk in Adults According to Family History. *JAMA Netw Open* **3**, e203959 (2020).

18. Patel, A. P., Wang, M., Kartoun, U., Ng, K. & Khera, A. V. Quantifying and Understanding the Higher Risk of Atherosclerotic Cardiovascular Disease Among South Asian Individuals. *Circulation* **144**, 410–422 (2021).
19. Lee, S. H., Goddard, M. E., Wray, N. R. & Visscher, P. M. A better coefficient of determination for genetic profile analysis. *Genet Epidemiol* **36**, 214–224 (2012).
20. Khera, A. V. *et al.* Genome-wide polygenic scores for common diseases identify individuals with risk equivalent to monogenic mutations. *Nat. Genet.* **50**, 1219–1224 (2018).
21. Goff David C. *et al.* 2013 ACC/AHA Guideline on the Assessment of Cardiovascular Risk. *Circulation* **129**, S49–S73 (2014).

Genes & Health Study Acknowledgements

Current Genes & Health Research Team (in alphabetical order by surname): Shaheen Akhtar, Mohammad Anwar, Elena Arciero, Omar Asgar, Samina Ashraf, Saeed Bidi, Gerome Breen, James Broster, Raymond Chung, David Collier, Charles J Curtis, Shabana Chaudhary, Megan Clinch, Grainne Colligan, Panos Deloukas, Ceri Durham, Faiza Durrani, Fabiola Eto, Sarah Finer, Joseph Gafton, Ana Angel Garcia, Chris Griffiths, Joanne Harvey, Teng Heng, Sam Hodgson, Qin Qin Huang, Matt Hurles, Karen A Hunt, Shapna Hussain, Kamrul Islam, Vivek Iyer, Ben Jacobs, Ahsan Khan, Cath Lavery, Sang Hyuck Lee, Robin Lerner, Daniel MacArthur, Daniel Malawsky, Hilary Martin, Dan Mason, Rohini Mathur, Mohammed Bodrul Mazid, John McDermott, Caroline Morton, Bill Newman, Elizabeth Owor, Asma Qureshi, Samiha Rahman, Shwetha Ramachandrappa, Mehru Reza, Jessry Russell, Nishat Safa, Miriam Samuel, Michael Simpson, John Solly, Marie Spreckley. Daniel Stow, Michael Taylor, Richard C Trembath, Karen Tricker, Nasir Uddin, David A van Heel, Klaudia Walter, Caroline Winckley, Suzanne Wood, John Wright, Julia Zollner.

VA Million Veteran Program:

MVP Program Office

- Sumitra Muralidhar, Ph.D., Program Director
  US Department of Veterans Affairs, 810 Vermont Avenue NW, Washington, DC 20420
- Jennifer Moser, Ph.D., Associate Director, Scientific Programs
  US Department of Veterans Affairs, 810 Vermont Avenue NW, Washington, DC 20420
- Jennifer E. Deen, B.S., Associate Director, Cohort & Public Relations
  US Department of Veterans Affairs, 810 Vermont Avenue NW, Washington, DC 20420

MVP Executive Committee

- Co-Chair: Philip S. Tsao, Ph.D.
  VA Palo Alto Health Care System, 3801 Miranda Avenue, Palo Alto, CA 94304
- Co-Chair: Sumitra Muralidhar, Ph.D.
  US Department of Veterans Affairs, 810 Vermont Avenue NW, Washington, DC 20420
- J. Michael Gaziano, M.D., M.P.H.
  VA Boston Healthcare System, 150 S. Huntington Avenue, Boston, MA 02130
- Elizabeth Hauser, Ph.D.
  Durham VA Medical Center, 508 Fulton Street, Durham, NC 27705
- Amy Kilbourne, Ph.D., M.P.H.
  VA HSR&D, 2215 Fuller Road, Ann Arbor, MI 48105
- Shiuh-Wen Luoh, M.D., Ph.D.
  VA Portland Health Care System, 3710 SW US Veterans Hospital Rd, Portland, OR 97239
- Michael Matheny, M.D., M.S., M.P.H.
  VA Tennessee Valley Healthcare System, 1310 24th Ave. South, Nashville, TN 37212
- Dave Oslin, M.D.
  Philadelphia VA Medical Center, 3900 Woodland Avenue, Philadelphia, PA 19104

MVP Co-Principal Investigators

- J. Michael Gaziano, M.D., M.P.H.
  VA Boston Healthcare System, 150 S. Huntington Avenue, Boston, MA 02130
- Philip S. Tsao, Ph.D.
  VA Palo Alto Health Care System, 3801 Miranda Avenue, Palo Alto, CA 94304

MVP Core Operations

- Lori Churby, B.S., Director, MVP Regulatory Affairs
  VA Palo Alto Health Care System, 3801 Miranda Avenue, Palo Alto, CA 94304
- Stacey B. Whitbourne, Ph.D., Director, MVP Cohort Management
  VA Boston Healthcare System, 150 S. Huntington Avenue, Boston, MA 02130
- Jessica V. Brewer, M.P.H., Director, MVP Recruitment & Enrollment
  VA Boston Healthcare System, 150 S. Huntington Avenue, Boston, MA 02130
- Shahpoor (Alex) Shayan, M.S., Director, MVP Recruitment and Enrollment Informatics
  VA Boston Healthcare System, 150 S. Huntington Avenue, Boston, MA 02130
- Luis E. Selva, Ph.D., Executive Director, MVP Biorepositories

VA Boston Healthcare System, 150 S. Huntington Avenue, Boston, MA 02130
- Saiju Pyarajan Ph.D., Director, Data and Computational Sciences
  VA Boston Healthcare System, 150 S. Huntington Avenue, Boston, MA 02130
- Kelly Cho, M.P.H, Ph.D., Director, MVP Phenomics Data Core
  VA Boston Healthcare System, 150 S. Huntington Avenue, Boston, MA 02130
- Scott L. DuVall, Ph.D., Director, VA Informatics and Computing Infrastructure (VINCI)
  VA Salt Lake City Health Care System, 500 Foothill Drive, Salt Lake City, UT 84148
- Mary T. Brophy M.D., M.P.H., Director, VA Central Biorepository
  VA Boston Healthcare System, 150 S. Huntington Avenue, Boston, MA 02130
- MVP Coordinating Centers
  - MVP Coordinating Center, Boston - J. Michael Gaziano, M.D., M.P.H.
    VA Boston Healthcare System, 150 S. Huntington Avenue, Boston, MA 02130
  - MVP Coordinating Center, Palo Alto – Philip S. Tsao, Ph.D.
    VA Palo Alto Health Care System, 3801 Miranda Avenue, Palo Alto, CA 94304
  - MVP Information Center, Canandaigua – Brady Stephens, M.S.
    Canandaigua VA Medical Center, 400 Fort Hill Avenue, Canandaigua, NY 14424
  - Cooperative Studies Program Clinical Research Pharmacy Coordinating Center, Albuquerque – Todd Connor, Pharm.D.; Dean P. Argyres, B.S., M.S.
    New Mexico VA Health Care System, 1501 San Pedro Drive SE, Albuquerque, NM 87108

MVP Publications and Presentations Committee

- Co-Chair: Tim Assimes, M.D.
  VA Palo Alto Health Care System, 3801 Miranda Avenue, Palo Alto, CA 94304
- Co-Chair: Adriana Hung, M.D.
  VA Tennessee Valley Healthcare System, 1310 24th Ave. South, Nashville, TN 37212
- Co-Chair: Henry Kranzler, M.D.
  Philadelphia VA Medical Center, 3900 Woodland Avenue, Philadelphia, PA 19104

MVP Local Site Investigators

- Samuel Aguayo, M.D., Phoenix VA Health Care System
  650 E. Indian School Road, Phoenix, AZ 85012
- Sunil Ahuja, M.D., South Texas Veterans Health Care System
  7400 Merton Minter Boulevard, San Antonio, TX 78229
- Kathrina Alexander, M.D., Veterans Health Care System of the Ozarks
  1100 North College Avenue, Fayetteville, AR 72703
- Xiao M. Androulakis, M.D., Columbia VA Health Care System
  6439 Garners Ferry Road, Columbia, SC 29209
- Prakash Balasubramanian, M.D., William S. Middleton Memorial Veterans Hospital
  2500 Overlook Terrace, Madison, WI 53705
- Zuhair Ballas, M.D., Iowa City VA Health Care System
  601 Highway 6 West, Iowa City, IA 52246-2208
- Jean Beckham, Ph.D., Durham VA Medical Center
  508 Fulton Street, Durham, NC 27705
- Sujata Bhushan, M.D., VA North Texas Health Care System
  4500 S. Lancaster Road, Dallas, TX 75216
- Edward Boyko, M.D., VA Puget Sound Health Care System
  1660 S. Columbian Way, Seattle, WA 98108-1597
- David Cohen, M.D., Portland VA Medical Center
  3710 SW U.S. Veterans Hospital Road, Portland, OR 97239

- Louis Dellitalia, M.D., Birmingham VA Medical Center
  700 S. 19th Street, Birmingham AL 35233
- L. Christine Faulk, M.D., Robert J. Dole VA Medical Center
  5500 East Kellogg Drive, Wichita, KS 67218-1607
- Joseph Fayad, M.D., VA Southern Nevada Healthcare System
  6900 North Pecos Road, North Las Vegas, NV 89086
- Daryl Fujii, Ph.D., VA Pacific Islands Health Care System
  459 Patterson Rd, Honolulu, HI 96819
- Saib Gappy, M.D., John D. Dingell VA Medical Center
  4646 John R Street, Detroit, MI 48201
- Frank Gesek, Ph.D., White River Junction VA Medical Center
  163 Veterans Drive, White River Junction, VT 05009
- Jennifer Greco, M.D., Sioux Falls VA Health Care System
  2501 W 22nd Street, Sioux Falls, SD 57105
- Michael Godschalk, M.D., Richmond VA Medical Center
  1201 Broad Rock Blvd., Richmond, VA 23249
- Todd W. Gress, M.D., Ph.D., Hershel “Woody” Williams VA Medical Center
  1540 Spring Valley Drive, Huntington, WV 25704
- Samir Gupta, M.D., M.S.C.S., VA San Diego Healthcare System
  3350 La Jolla Village Drive, San Diego, CA 92161
- Salvador Gutierrez, M.D., Edward Hines, Jr. VA Medical Center
  5000 South 5th Avenue, Hines, IL 60141
- John Harley, M.D., Ph.D., Cincinnati VA Medical Center
  3200 Vine Street, Cincinnati, OH 45220
- Kimberly Hammer, Ph.D., Fargo VA Health Care System
  2101 N. Elm, Fargo, ND 58102
- Mark Hamner, M.D., Ralph H. Johnson VA Medical Center
  109 Bee Street, Mental Health Research, Charleston, SC 29401
- Adriana Hung, M.D., M.P.H., VA Tennessee Valley Healthcare System
  1310 24th Avenue, South Nashville, TN 37212
- Robin Hurley, M.D., W.G. (Bill) Hefner VA Medical Center
  1601 Brenner Ave, Salisbury, NC 28144
- Pran Iruvanti, D.O., Ph.D., Hampton VA Medical Center
  100 Emancipation Drive, Hampton, VA 23667
- Frank Jacono, M.D., VA Northeast Ohio Healthcare System
  10701 East Boulevard, Cleveland, OH 44106
- Darshana Jhala, M.D., Philadelphia VA Medical Center
  3900 Woodland Avenue, Philadelphia, PA 19104
- Scott Kinlay, M.B.B.S., Ph.D., VA Boston Healthcare System
  150 S. Huntington Avenue, Boston, MA 02130
- Jon Klein, M.D., Ph.D., Louisville VA Medical Center
  800 Zorn Avenue, Louisville, KY 40206
- Michael Landry, Ph.D., Southeast Louisiana Veterans Health Care System
  2400 Canal Street, New Orleans, LA 70119
- Peter Liang, M.D., M.P.H., VA New York Harbor Healthcare System
  423 East 23rd Street, New York, NY 10010
- Suthat Liangpunsakul, M.D., M.P.H., Richard Roudebush VA Medical Center
  1481 West 10th Street, Indianapolis, IN 46202
- Jack Lichy, M.D., Ph.D., Washington DC VA Medical Center
  50 Irving St, Washington, D. C. 20422
- C. Scott Mahan, M.D., Charles George VA Medical Center
  1100 Tunnel Road, Asheville, NC 28805
- Ronnie Marrache, M.D., VA Maine Healthcare System
  1 VA Center, Augusta, ME 04330

- Stephen Mastorides, M.D., James A. Haley Veterans' Hospital
  13000 Bruce B. Downs Blvd, Tampa, FL 33612
- Elisabeth Mates M.D., Ph.D., VA Sierra Nevada Health Care System
  975 Kirman Avenue, Reno, NV 89502
- Kristin Mattocks, Ph.D., M.P.H., Central Western Massachusetts Healthcare System
  421 North Main Street, Leeds, MA 01053
- Paul Meyer, M.D., Ph.D., Southern Arizona VA Health Care System
  3601 S 6th Avenue, Tucson, AZ 85723
- Jonathan Moorman, M.D., Ph.D., James H. Quillen VA Medical Center
  Corner of Lamont & Veterans Way, Mountain Home, TN 37684
- Timothy Morgan, M.D., VA Long Beach Healthcare System
  5901 East 7th Street Long Beach, CA 90822
- Maureen Murdoch, M.D., M.P.H., Minneapolis VA Health Care System
  One Veterans Drive, Minneapolis, MN 55417
- James Norton, Ph.D., VA Health Care Upstate New York
  113 Holland Avenue, Albany, NY 12208
- Olaoluwa Okusaga, M.D., Michael E. DeBakey VA Medical Center
  2002 Holcombe Blvd, Houston, TX 77030
- Kris Ann Oursler, M.D., Salem VA Medical Center
  1970 Roanoke Blvd, Salem, VA 24153
- Ana Palacio, M.D., M.P.H., Miami VA Health Care System
  1201 NW 16th Street, 11 GRC, Miami FL 33125
- Samuel Poon, M.D., Manchester VA Medical Center
  718 Smyth Road, Manchester, NH 03104
- Emily Potter, Pharm.D., VA Eastern Kansas Health Care System
  4101 S 4th Street Trafficway, Leavenworth, KS 66048
- Michael Rauchman, M.D., St. Louis VA Health Care System
  915 North Grand Blvd, St. Louis, MO 63106
- Richard Servatius, Ph.D., Syracuse VA Medical Center
  800 Irving Avenue, Syracuse, NY 13210
- Satish Sharma, M.D., Providence VA Medical Center
  830 Chalkstone Avenue, Providence, RI 02908
- River Smith, Ph.D., Eastern Oklahoma VA Health Care System
  1011 Honor Heights Drive, Muskogee, OK 74401
- Peruvemba Sriram, M.D., N. FL/S. GA Veterans Health System
  1601 SW Archer Road, Gainesville, FL 32608
- Patrick Strollo, Jr., M.D., VA Pittsburgh Health Care System
  University Drive, Pittsburgh, PA 15240
- Neeraj Tandon, M.D., Overton Brooks VA Medical Center
  510 East Stoner Ave, Shreveport, LA 71101
- Philip Tsao, Ph.D., VA Palo Alto Health Care System
  3801 Miranda Avenue, Palo Alto, CA 94304-1290
- Gerardo Villareal, M.D., New Mexico VA Health Care System
  1501 San Pedro Drive, S.E. Albuquerque, NM 87108
- Agnes Wallbom, M.D., M.S., VA Greater Los Angeles Health Care System
  11301 Wilshire Blvd, Los Angeles, CA 90073
- Jessica Walsh, M.D., VA Salt Lake City Health Care System
  500 Foothill Drive, Salt Lake City, UT 84148
- John Wells, Ph.D., Edith Nourse Rogers Memorial Veterans Hospital
  200 Springs Road, Bedford, MA 01730
- Jeffrey Whittle, M.D., M.P.H., Clement J. Zablocki VA Medical Center
  5000 West National Avenue, Milwaukee, WI 53295
- Mary Whooley, M.D., San Francisco VA Health Care System
  4150 Clement Street, San Francisco, CA 94121

- Allison E. Williams, N.D., Ph.D., R.N, Bay Pines VA Healthcare System
  10,000 Bay Pines Blvd Bay Pines, FL 33744
- Peter Wilson, M.D., Atlanta VA Medical Center
  1670 Clairmont Road, Decatur, GA 30033
- Junzhe Xu, M.D., VA Western New York Healthcare System
  3495 Bailey Avenue, Buffalo, NY 14215-1199
- Shing Shing Yeh, Ph.D., M.D., Northport VA Medical Center
  79 Middleville Road, Northport, NY 11768
