# Supplementary material for: A multi-ancestry polygenic risk score improves risk prediction for coronary artery disease
Source: Nat Med. 2023 Jul 6;29(7):1793–803. doi: 10.1038/s41591-023-02429-x (PMC10353935; doi:10.1038/s41591-023-02429-x)
Supplement: Supplementary file 2 — Reporting Summary [file 41591_2023_2429_MOESM2_ESM.pdf]

nature portfolio

Corresponding author(s): Amit V. Khera and Minxian Wang

Last updated by author(s): May 22, 3023

Reporting Summary

Nature Portfolio wishes to improve the reproducibility of the work that we publish. This form provides structure for consistency and transparency in reporting. For further information on Nature Portfolio policies, see our Editorial Policies and the Editorial Policy Checklist.

Statistics

For all statistical analyses, confirm that the following items are present in the figure legend, table legend, main text, or Methods section.

n/a	Confirmed	
☐	☒	The exact sample size (*n*) for each experimental group/condition, given as a discrete number and unit of measurement
☐	☒	A statement on whether measurements were taken from distinct samples or whether the same sample was measured repeatedly
☐	☒	The statistical test(s) used AND whether they are one- or two-sided<br>*Only common tests should be described solely by name; describe more complex techniques in the Methods section.*
☐	☒	A description of all covariates tested
☐	☒	A description of any assumptions or corrections, such as tests of normality and adjustment for multiple comparisons
☐	☒	A full description of the statistical parameters including central tendency (e.g. means) or other basic estimates (e.g. regression coefficient) AND variation (e.g. standard deviation) or associated estimates of uncertainty (e.g. confidence intervals)
☐	☒	For null hypothesis testing, the test statistic (e.g. *F*, *t*, *r*) with confidence intervals, effect sizes, degrees of freedom and *P* value noted<br>*Give P values as exact values whenever suitable.*
☒	☐	For Bayesian analysis, information on the choice of priors and Markov chain Monte Carlo settings
☒	☐	For hierarchical and complex designs, identification of the appropriate level for tests and full reporting of outcomes
☒	☐	Estimates of effect sizes (e.g. Cohen's *d*, Pearson's *r*), indicating how they were calculated

Our web collection on statistics for biologists contains articles on many of the points above.

Software and code

Policy information about availability of computer code

Data collection	No custom software or algorithms were developed for data collection.
Data analysis	We used following softwares for imputation, handling the genetic and phenotypic data: Plink 1.9 and 2.0; BCFtools 1.7 and 1.9; R-3.5; LDpred2 (version 1.7.1); bgenix (version 1.1.7); MASS package(version: 7.3-51.4); Data mash (version 1.2.0). For data transformations, visualization and plotting of the results, we used R 3.5 and 3.6, including packages rcompanion (version 2.4.30), nricens (version 1.6), ggplot2 (version 3.4.2), survminer (version 0.4.9).

For manuscripts utilizing custom algorithms or software that are central to the research but not yet described in published literature, software must be made available to editors and reviewers. We strongly encourage code deposition in a community repository (e.g. GitHub). See the Nature Portfolio guidelines for submitting code & software for further information.

Data

Policy information about availability of data

All manuscripts must include a data availability statement. This statement should provide the following information, where applicable:

- Accession codes, unique identifiers, or web links for publicly available datasets
- A description of any restrictions on data availability
- For clinical datasets or third party data, please ensure that the statement adheres to our policy

All data are made available from the UK Biobank (https://www.ukbiobank.ac.uk/enable-your-research/apply-for-access) to researchers from universities and other

institutions with genuine research inquiries following institutional review board and UK Biobank approval. This research was conducted using the UK Biobank resource under Application Number 7089 and approved by the Mass General Brigham institutional review board. The genome-wide association data supporting the findings of this study are publicly available in Biobank Japan (http://jenger.riken.jp/en/result), FinnGen (https://www.finngen.fi/en/access_results), AGEN T2D (https://kp4cd.org/index.php/node/309), GIANT (https://portals.broadinstitute.org/collaboration/giant/), Global Lipids Genetics Consortium (http://csg.sph.umich.edu/willer/public/glgc-lipids2021), Million Veterans Program (via dbGaP at https://ftp.ncbi.nlm.nih.gov/dbgap/studies/, under accession number phs001672), and upon request from CARDIoGRAMplusC4D (http://www.cardiogramplusc4d.org/data-downloads/), MEGASTROKE (http://megastroke.org/download.html), and Genes & Health (https://www.genesandhealth.org/research/scientific-data-downloads). The full GPSMult weights will be made available in the Polygenic Score Catalog through accession ID PGS003725.

Research involving human participants, their data, or biological material

Policy information about studies with human participants or human data. See also policy information about sex, gender (identity/presentation), and sexual orientation and race, ethnicity and racism.

Reporting on sex and gender

Our manuscript is compliant with the journal's policy on sex and gender reporting and sex was carefully considered in our study design. Sex was self-reported by participants when they registered for the UK Biobank, Million Veteran Program, and Genes & Health Studies. Our models include sex as on of the variables used to estimate the risk of coronary artery disease. We also report performance of GPSMult stratified by sex in the extended data.

Reporting on race, ethnicity, or other socially relevant groupings

Our manuscript uses continental ancestry to specify population subgroups to standardize reporting used in UK- and US-based cohorts. In the UK Biobank, individuals who self-reported as White, White British, Irish or other White background were grouped as European ancestry; individuals who self-reported as Asian, Asian British, Indian, Pakistani, Bangladeshi or Any other Asian background with country of origin being in South Asia were grouped as South Asian ancestry; individuals who self-reported as Black, Black British, Caribbean, African, or any other Black background were grouped as African ancestry, and individuals who self-reported as Chinese or Any other Asian background with country of origin being in East Asia were grouped as East Asian ancestry. In Million Veteran Program, individuals who self-reported as non-Hispanic White were grouped as European ancestry; individuals who self-reported as non-Hispanic Black were grouped as African ancestry, and individuals who self-reported as Hispanic were grouped as Hispanic ancestry. Individuals in Genes & Health who self-reported as Asian, Asian British, Pakistani, or Bangladeshi were grouped as South Asian. All analyses were carried out in subgroups stratified by continental ancestry and the first 10 principal components of genetic ancestry were used as covariates in all regression analyses.

Population characteristics

These scores were validated within the non-overlapping UK Biobank cohort in 116,645 individuals of European ancestry (mean age 57.5 yr, 47.5% male, 4412 CAD cases and 112,237 controls) and then tested in an independent study population in the UK Biobank [African ancestry N=7281 (mean age 52.4, 43.5% male, 124 CAD cases, 7157 controls), East Asian ancestry N=1464 (mean age 53, 37.2% male, 22 cases, 1442 controls), European ancestry N=308264 (mean age 57.3 yr, 45.6% male, 10492 cases, 297772 controls), and South Asian ancestry N=8982 (mean age 53.8, 54.1% male, 542 CAD cases and 8440 controls)], Million Veteran Program [African ancestry N=33096 (mean age 56.1, 84.1% male, 4831 CAD cases, 28265 controls), European ancestry N=124467 (mean age 60.8, 91.3% male, 29171 CAD cases, 95296 controls), Hispanic ancestry N=16433, mean age 51.9, 87.7% male, 2140 CAD cases, 14293 controls], and Genes & Health Study (South Asian ancestry N=16874, mean age 40.6, 45.9% male, 853 CAD cases, 16021 controls).

Recruitment

The UK Biobank is a prospective national biobank study that enrolled about 500,000 middle-aged adult participants between 2006 and 2010. Case definitions were based on self-report, hospitalization records, and death registry records. Participants within the Million Veteran Program were recruited from more than 75 Veteran Affairs Medical Centers nationwide since 2011, with >885,000 individuals currently enrolled. Each participant has consented to linkage to their electronic medical record, wherein ICD9/10 diagnosis codes, Current Procedural Terminology (CPT) codes, clinical laboratory measurements, and reports of diagnostic imaging modalities are available. Participants were also asked to complete baseline and lifestyle questionnaires to further augment data contained in the electronic health record. Genes & Health is a UK-based cohort of over 48,000 British Pakistani and Bangladeshi individuals recruited and consented for lifelong electronic health record access and genetic analysis. Medical records are linked to ICDl0, OPCS, and SNOMED diagnosis and procedural codes across inpatient and hospital settings as well as clinical laboratory measurements, and a baseline questionnaire.

Ethics oversight

Informed consent was obtained from all participants. This research was approved by the Mass General Brigham institutional review board (protocol 2021P002228).

Note that full information on the approval of the study protocol must also be provided in the manuscript.

Field-specific reporting

Please select the one below that is the best fit for your research. If you are not sure, read the appropriate sections before making your selection.

☒ Life sciences ☐ Behavioural & social sciences ☐ Ecological, evolutionary & environmental sciences

For a reference copy of the document with all sections, see nature.com/documents/nr-reporting-summary-flat.pdf

Life sciences study design

All studies must disclose on these points even when the disclosure is negative.

Sample size

These scores were validated within the non-overlapping UK Biobank cohort in 116,645 individuals of European ancestry and then tested in an independent study population in the UK Biobank (African ancestry N=7281, East Asian ancestry N=1464, European ancestry N=308264, and

	South Asian ancestry N=8982), Million Veteran Program (African ancestry N=33096, European ancestry N=124467, Hispanic ancestry N=16433), and Genes & Health Study (South Asian ancestry N=16874). Sample size for the UK Biobank, Milion Veteran project, and Genes & Health Study were determined by the number of subjects who were genotyped and passed quality control. These sample sizes provide adequate power to detect significant associations of GPSMult and previously published polygenic scores for coronary artery disease with modest effect.
Data exclusions	Individuals were excluded based on excessive DNA contamination, low target base coverage, putative sex chromosome aneupoloidy, outliers, for heterozygosity, or low genotyping array call rate. For each pair of related individuals (second-degree or closer), one was removed. Additionally, participants who withdrew consent following initial enrollment were excluded.
Replication	Careful steps were taken to obtain polygenic risk score weights from independent cohorts. After training in a hold out of the White British UK Biobank participants, for score validation, the analyses of polygenic risk score associations with coronary artery disease were replicated using the exact same procedure in 4 ancestry-stratified cohorts of the remaining individuals in the UK Biobank (African, East Asian, European, and South Asian ancestry), 3 ancestry-stratified cohorts in the Million Veteran Program (African, European, and Hispanic ancestry), and in the Genes & Health Study (South Asian ancestry).
Randomization	Not applicable as this is an observational study.
Blinding	Not applicable as this is an observational study.

Reporting for specific materials, systems and methods

We require information from authors about some types of materials, experimental systems and methods used in many studies. Here, indicate whether each material, system or method listed is relevant to your study. If you are not sure if a list item applies to your research, read the appropriate section before selecting a response.

Materials & experimental systems

n/a	Involved in the study
☒	☐ Antibodies
☒	☐ Eukaryotic cell lines
☒	☐ Palaeontology and archaeology
☒	☐ Animals and other organisms
☒	☐ Clinical data
☒	☐ Dual use research of concern
☒	☐ Plants

Methods

n/a	Involved in the study
☒	☐ ChIP-seq
☒	☐ Flow cytometry
☒	☐ MRI-based neuroimaging
